# Metabolomic mechanism and pharmacodynamic material basis of Buxue Yimu pills in the treatment of anaemia in women of reproductive age

**DOI:** 10.3389/fphar.2022.962850

**Published:** 2023-01-10

**Authors:** Guo Ying-ying, Wang Yan-fang, Deng Yan, Zhang Su-ying, Liu Dong, Luo Bin, Wang Xue, Deng Miao, Ma Rui-lin, Liu Xiao-hui, Jiao Yu-pei, Sun Ai-jun

**Affiliations:** ^1^ Department of Obstetrics and Gynecology, Peking Union Medical College Hospital, Chinese Academy of Medical Sciences and Peking Union Medical College, Beijing, China; ^2^ Department of Obstetrics and Gynecology, The Second Affiliated Hospital of Hunan University of Chinese Medicine, Changsha, China; ^3^ Key Laboratory of Birth Defects and Related Diseases of Women and Children, West China Second Hospital, Sichuan University, Chengdu, China; ^4^ Ministry of Education, Sichuan University, Chengdu, China; ^5^ Department of Obstetrics and Gynecology, Hangzhou Women’s Hospital (Hangzhou Maternity and Child Healthcare Hospital), Hangzhou, China; ^6^ School of Life Sciences, Tsinghua University, Beijing, China; ^7^ National Protein Science Technology Center, Tsinghua University, Beijing, China

**Keywords:** metabolomic, pharmacodynamic, anaemia, Buxue Yimu pills, iron stasis

## Abstract

**Objective:** To explore the pharmacological basis and mechanism of Buxue Yimu pills (BYP) in the treatment of anaemia in women from the perspective of metabolomics and network analysis.

**Materials and Methods:** Forty-six women of reproductive age with haemoglobin 70–110 g/L were recruited. Blood samples were collected before and after 4 weeks of oral BYP treatment to assess the changes in haemoglobin, coagulation function, and iron metabolism indices. An integrated analysis of metabolomics (liquid chromatography mass spectrometry) and network analysis was performed to identify the potential pharmacodynamic mechanisms of BYP.

**Results:** After BYP treatment, the haemoglobin level of patients significantly increased from 93.67 ± 9.77 g/L to 109.28 ± 12.62 g/L (*p* < 0.01), while no significant changes were found in iron metabolism and coagulation-related indicators. A total of 22 differential metabolites were identified after metabolomics analysis, which were mainly related to the inhibition of inflammation and oxidative stress. Integrating pharmacodynamics and metabolomics, a network of drug-active components-targets-metabolic pathways-metabolomics was established. Acetylcholinesterase, phospholipase A2 group IIA, and phospholipase A2 group IVA may be the most promising therapeutic targets.

**Conclusion:** BYP can inhibit inflammation and oxidative stress as well as promote haematopoiesis, potentially improving anaemia.

## 1 Introduction

Anaemia is thought to affect 29% and 38% of non-pregnant and pregnant women, respectively (Benson et al., 2021). Its high prevalence and recurrent nature have significantly affected the work and life of women of childbearing age. Gynaecological disorders often include recurrent menorrhagia, chronic postoperative and postpartum bleeding, and abnormal uterine bleeding caused by adenomyosis, fibroids, and endometrial hyperplasia (Breymann and Auerbach, 2017). Iron deficiency anaemia is often considered to be the most common single cause of anaemia, with multi-country meta-analyses worldwide showing that it accounts for 34%–50% of cases, mainly due to a lack of iron supply, inadequate bioavailability, or blood loss. Other causes include nutritional anaemia such as vitamin/folic acid deficiency and drug-related anaemia.

The goal of the World Health Organization is to halve the prevalence of anaemia in women of reproductive age between 2010 and 2025 (Young, 2018). Anaemia treatment decisions are based on a combination of factors, such as the patient age, sex, cause, severity, symptoms, and time available for treatment. Available treatment modalities include iron, other nutritional drugs such as vitamin B12/folic acid, erythropoietin (EPO), or surgical control supplemented by blood transfusions if the patient is haemodynamically unstable and severely anaemic (Sato and Yanagita, 2013). Oral iron is convenient, inexpensive, and effective but is prone to cause gastrointestinal discomfort, muscle pain, and urticaria. Compliance and tolerability are common factors that limit its efficacy (Pavord et al., 2020). Regardless of readiness, nearly 20% of women discontinue oral iron therapy (Ortiz et al., 2011). Intravenous iron is often administered when oral therapy is ineffective or inhibits absorption and can bypass the hepcidin block, which limits oral iron absorption (Weiss et al., 2019). However, the incidence of adverse reactions is also 1%–5% (Breymann and Auerbach, 2017), which commonly include nausea, headache, hypertension, flushing, and injection site reactions. Alternatively, other parenteral alternatives to oral iron, such as iron polymermaltose, gluconate iron, and low molecular weight iron, are available, but further research is needed to validate evidence-based medicine in large samples. Despite the benefits of iron supplementation, the long-term biological effects of iron, including oxygen radical production, will lead to an increased incidence of infectious diseases.

As a result, western medicine alone often has various limitations, while traditional Chinese medicine (TCM) prescriptions often have the distinct advantage of fewer side effects in the treatment of multifactorial diseases because of their natural multi-active ingredient composition, multi-pathway targets, and holistic treatment bias (Wang et al., 2016). TCM is considered as a complementary and alternative form of medicine in the Chinese healthcare system (Feng et al., 2021). It is often used together with western medicine to reduce side effects or toxicity or to obtain additional pharmacological effects, but attention should also be paid to the presence of herbal drug interactions due to the inhibition or induction of cytochrome enzymes (Wang, 2015).

Based on the syndrome differentiation and treatment theory of TCM, qi and blood deficiency are the causes of angiogenesis disorders and blood flow disturbance. Qi-reinforced, blood-activating, and blood-stasis-removing drugs have huge treatment potential (Zhang et al., 2022). Numerous herbal formulations and extracts have been shown to promote angiogenesis, improve blood circulation, and clear blood stasis (Bu et al., 2020; Ni et al., 2021). Buxue Yimu pills (BYP) have attracted attention for replenishing qi and blood and for dispelling blood stasis. According to the 2015 edition of Chinese Pharmacopeia, it is mainly composed of five ingredients namely, *Angelicae Sinensis* Radix [ASR, the dried root of *Angelica sinensis* (Oliv.) Diels], *Astragali* Radix (AR, the dried root of Astragalus mongholicus Bunge), Asini Corii Colla (ACC, a product of the hide of *Equus asinus L.*), *Leonurus cardiaca L.* (LC), Citri Reticulatae Pericarpium (CRP, dried citrus peel from Citrus × *aurantium L.*), in which the bioactive substance has been analysed (Zhang et al., 2020). It is widely used for the treatment of gynaecological diseases, postpartum or abortion complications, and postpartum abdominal pain. Modern pharmacological evidence suggests that it can effectively increase haemoglobin levels, promote endometrial repair, and improve metabolic disorders. However, the specific effects and molecular mechanisms of its pharmacological effects in improving anaemia are not clear.

Metabolomics is a well-established histological technique in the post-genomic era and has long been used in TCM, focusing on identifying endogenous low-molecular metabolites in response to internal and external features and providing clues about its molecular basis and mechanisms of action. It can play an active role in disease control rate assessment and elucidation of the mechanisms of action of TCM (Yang and Lao, 2019). On the other hand, network analysis, a new tool based on data mining and network construction, has been used to thoroughly investigate the active ingredients, potential targets, and underlying theoretical mechanisms of TCM. Therefore, in our study, we explored the mechanism of BYP in anaemia by integrating metabolomics and network pharmacological approaches.

## 2 Materials and methods

### 2.1 Clinical sample design

Forty-six women of reproductive age caused by deficiency of Qi and Blood, combined with blood stasis syndrome, were investigated in this study, which is similar to that of chronic anemia and hemorrhagic anemia in Western medicine. In terms of clinical parameters, patients with a haemoglobin of 70–110 g/L and defined gynecological cause for polyps, fibroids and so on were included. Patients with uncontrolled bleeding, abnormal liver and kidney functions, malignancy, psychiatric disorders, or pregnancy were excluded. All patients signed an informed consent form. The study was approved by the PUMCH Ethics Committee (No. ZS-1254) and registered with the National Institutes of Health (registration No. NCT03232554, www.clinicaltrials.gov). This study was conducted in accordance with the Declaration of Helsinki of the World Medical Association.

### 2.2 Intervention assessment

Participants received BYP (24 g, twice a day) through oral administration for 4 weeks. BYP (Zhuzhou Qianjin Pharmaceutical Co., Ltd., Hunan, China, CFDA approval number: Z20090602, batch number: 20170403) were stored in bags (12 g/bag) and consisted of five ingredients (See Table 1 for details of composition). According to the high-performance liquid chromatography (HPLC) method, the content of ferulic acid (C10H10O4) was ensured to be not less than 1 mg for each bag (Pharmacopoeia Commission, 2005).

**TABLE 1:** Standard formulation of Buxue Yimu Pills (1000g).

Chinese pinyin name	Scientific name	Proportion (g)
Danggui	*Angelicae Sinensis Radix*	416.7
Huangqi	*Astragali Radix*	416.7
Ejiao	*Asini Corii Colla*	125
Yimucao	*Leonurus cardiaca L*	625
Chenpi	*Citri Reticulatae Pericarpium*	12.5

Venous blood samples were collected before and after treatment, and whole blood cell count, coagulation function, liver and kidney function, and iron metabolism-related indices, including the serum iron, serum ferritin, and total iron binding capacity, were assessed according to routine laboratory methods. Side-effects and adverse events were monitored.

### 2.3 Metabolomics research

#### 2.3.1 Metabolite extraction

Blood samples of 10 patients were collected for metabolomics analysis by liquid chromatography mass spectrometry/mass spectrometry (LC-MS/MS) at the Facility Center of Metabolomics and Lipidomics at the Technology Center for Protein Sciences of Tsinghua University. Briefly, 50 μL serum sample thawed at room temperature was mixed with a 450 μL methanol: acetonitrile (1:1) preparation (containing internal standard propranolol 50 ng/mL), which was then vortexed for 30 s and centrifuged at 13,000 r/min for 15 min. Afterward, an aliquot of 5 μL supernatant was taken and directly injected for analysis.

Furthermore, quality control (QC) samples were prepared by mixing equal volumes of extraction of all samples. The volume of each QC was the same as that of the sample. Since QC samples contained the data of every sample, they were applied to the stability verification of the LC-MS/MS system.

#### 2.3.2 LC-MS/MS analysis

LC-MS was performed on an Ultimate 3000-VelosPro system equipped with a binary solvent delivery manager and a sample manager, coupled with a QE Orbitrap Mass Spectrometer equipped with an electrospray interface (Thermo Fisher Scientific, United States).

First, an ACQUITY BEH C18 column (2.1 mm × 50 mm, 1.7 μm) was applied for the analytes, whose temperature was maintained at 30°C, with a flow rate of 0.25 mL/min. The analysis was performed with gradient elution using water/0.1% formic acid/2 mmoL/L ammonium formate (solvent A) and acetonitrile (solvent B), which were programmed as follows: 95% A from 0 to 1 min; 95%–40% A from 1 to 5 min; 40%–0% A from 5 to 8 min; 0% A from 8 to 11 min; 0%–40% A from 11 to 14 min; 40%–95% A from 14 to 15 min; 95% A from 15 to 18 min.

Next, data were collected in electrospray ionization (ESI) positive and negative ion modes, and the ESI voltage was set to 3.0 kV. The instrument settings were first an MS scan with 70,000 resolution, and then MS/MS scans of the top 10 most intense precursors with 17,500 resolutions. Data were acquired in the positive-ion mode in the mass range 70–1,050 m/z and in the negative-ion mode in the mass range 80–1,200 m/z.

#### 2.3.3 Metabolite identification

Metabolites were identified using Tracerfinder 3.2 (Thermo Fisher Scientific; California, United States) based on an internal MS/MS library, which was established using chemical standards or biological samples. Two levels of metabolite identification were achieved: one was confirmed by MS/MS and the other by mass matching of precursors. Metabolite candidates were selected from those identified by MS/MS with an LS score higher than 30 and those not confirmed by MS/MS. Our in-house software “MetaInt” was used for high-throughput data analysis.

### 2.4 Statistical analysis

#### 2.4.1 Clinical indicators analysis

SPSS 26.0 (IBM; Chicago, Illinois, United States) was used for statistical analysis. Normally distributed data are expressed as mean ± standard deviation, and the paired *t*-test was used to compare the differences before and after treatment. Non-normal distribution data are represented by the median +25–75 interquartile range, and the differences were evaluated using the Wilcoxon paired signed rank test. Statistical significance was defined as *p* < 0.05. Variable correlation analysis was performed using the Pearson correlation analysis or Spearman rank correlation.

#### 2.4.2 Metabolic profiling and pathway analysis

Data were normalized by a log10 transformation before statistical analysis. Multivariate statistics and pathway analysis were performed using MetaboAnalyst 3.0 (www.Metaboanalyst.ca), including principal component analysis (PCA), partial least squares discriminant analysis (PLSDA), and orthogonal partial least squares discriminant analysis (OPLSDA). The differential metabolites were selected according to variable importance (VIP) > 1 and *p*-value (*t*-test) < 0.05 out of Kyoto Encyclopedia of Genes and Genomes (KEGG) Pathway for enrichment analysis.

#### 2.4.3 Network construction

To further explain the changes in the metabolic spectrum and clarify the molecular mechanisms of BYP in the treatment of anaemia, we comprehensively applied network analysis. First, we obtained potential treatment targets of BYP bioactive compounds (oral bioavailability ≥30% and drug-likeness ≥ 0.18) by searching the TCMSP platform (http://lsp.nwu.edu.cn/tcmsp.php), which subsequently converted to target genes using Uniprot Knowledgebase (http://www.uniprot.org/). Second, anaemia targets were retrieved from the Online Mendelian Inheritance in Man (OMIM) database (https://www.omim.org/) and GeneCard Database (https://www.genecards.org/) without duplication using “gynecological anemia” as the keyword. Third, using the KEGG of the above differential metabolite enrichment analysis results, the related gene list was obtained from the KEGG2 webpage (http://www.genome.jp/kegg/kegg2.html). Finally, the drug-active components-targets-metabolic pathways-metabolomics network was established and visualised by Cytoscape software (http://cytoscape.org/).

## 3 Results

### 3.1 Baseline characteristics and pharmacodynamic results

All 46 subjects aged 24–52 years old (median age: 41 years) completed 4 weeks treatment. The median BMI was 23.1 kg/m^2^; 73% had a pregnancy history. The main causes of gynaecological anaemia were ovulation disorders (53.3%), surgical blood loss (17.8%), fibroids (11.1%), adenomyosis (8.9%), and polyps (8.9%).

As shown in Table 2, after 4 weeks of BYP treatment, the haemoglobin level of the patients increased significantly from 93.67 ± 9.77 g/L to 109.28 ± 12.62 g/L, red blood cell count from 3.790.53 × 1,012 g/L to 4.450.42 × 1,012 g/L, and haematocrit from 29.82 ± 2.81% to 34.52 ± 3.21% (*p* < 0.01).There were no significant changes in other blood cell counts, iron level, and coagulation indices (Table 2). Liver and renal function were also not significantly changed (*p* > 0.05). No adverse effects were recorded throughout the study.

**TABLE 2:** Comparison of serological indicators before and after BYP treatment in 46 patients.

	Before	After	*p*-value
Hb (g/L)	93.67 ± 9.77	109.28 ± 12.62*	<0.01**
MCV (fL)	78.91 ± 8.83	78.18 ± 8.57	0.368
MCH (pg)	25.04 ± 3.53	24.67 ± 3.28	0.261
MCHC (g/L)	314.18 ± 18.14	314.13 ± 11.56	0.986
RBC (×10^12^/L)	3.79 ± 0.53	4.45 ± 0.42*	<0.01**
HCT (%)	29.82 ± 2.81	34.52 ± 3.21*	<0.01**
Ret (%)	82.00 (59.88,100.75)	84.15 (62.50,97.28)	0.662
SI (μmol/L)	14.95 (7.63,32.15)	20.30 (11.35,32.50)	0.657
SF (μg/L)	9.45 (4.75,21.65)	8.80 (5.00,20.50)	0.175
TIBC (g/L)	346.50 (56.23,419.25)	381.00 (69.75,435.00)	0.071
PT	12.09 ± 0.97	11.91 ± 0.85	0.099
APTT	28.55 ± 4.22	28.44 ± 4.27	0.827
TT	21.29 ± 24.50	17.77 ± 0.88	0.342
FIB (g/L)	2.44 (2.17,2.73)	2.46 (2.23,2.97)	0.657
SCr (μmol/L)	56.93 ± 12.23	55.29 ± 12.78	0.341
BUN (mmol/L)	4.34 ± 1.00	4.62 ± 1.14	0.107
ALT	16.50 (12.00,20.75)	15.00 (12.00,21.00)	0.979

Notes: Data were presented as mean ± SD, for normally distributed parameters or as median (25% IQR, 75% IQR) for not normally distributed variables, *p < 0.05, **p < 0.01. Hb, hemoglobin; MCV, mean corpuscular volume; MCH, mean corpuscular hemoglobin; MCHC, mean corpuscular hemoglobin concentration; RBC, red blood cell count; HCT, hematocrit; Ret, reticulocyte; SI, serum iron; SF, serum ferritin; TIBC, total iron-binding capacity; PT, prothrombin time; APTT, activated partial thromboplastin time; TT, thrombin time; FIB, fibrinogen; SCr, serum creatinine; BUN, blood urea nitrogen; ALT, alanine aminotransferase

### 3.2 Metabolomics results

#### 3.2.1 Metabolomics analysis

**FIGURE 1:**
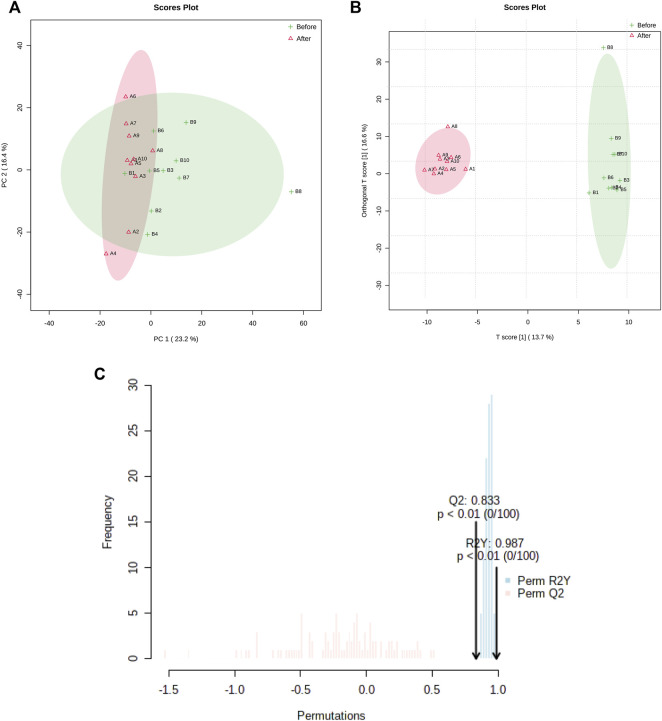
Metabolic profiling before and after treatment. **(A)**: PCA score plot. **(B)**: OPLSDA score plot. **(C)**: Validation of the model obtained from 100 permutation tests (R2Y = 0.987; Q2 = 0.833, p < 0.01).

Metabolomic data showed that 205 and 328 metabolites were identified and quantified in cationic and anionic modes, respectively. Two-dimensional PCA and OPLS-DA showed the separation and distribution of the metabolites before and after treatment (Figure 1). Model verification from 100 permutation tests showed the variance (R^2^Y = 0.987), predictivity (Q^2^ = 0.833), and *p*-values <0.01.

#### 3.2.2 Identification of differential metabolites and related metabolic pathways

**FIGURE 2:**
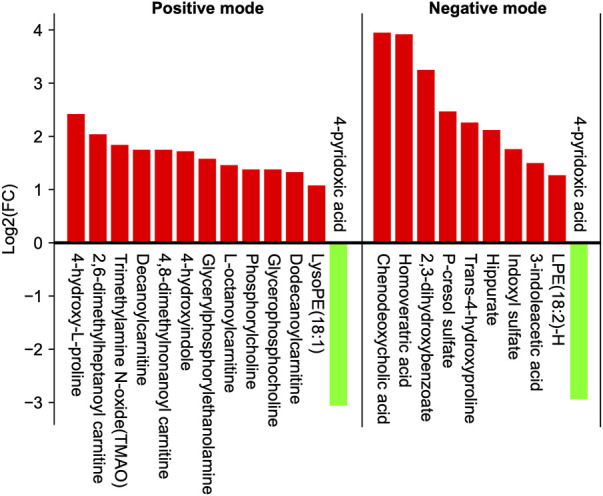
Changes in metabolome profile. Log 2(FC): log2 fold change, represents the values of differential metabolites fold change (after-/before-treatment) were transformed into log2-ratio. Green color indicates decreased concentration levels of metabolites, red color increased metabolite levels. Please refer to the Supplementary Table S1 for details.

According to the above metabolic data analysis methods, we identified 22 differential metabolites (13 in positive mode, 10 in negative mode, and one in both modes; Figure 2; Table 3). Supplementary Material contains the secondary mass spectrogram of each metabolite. After the BYP treatment, the concentrations of most differential metabolites increased, including chenodeoxycholic acid (fold change: 15.47, VIP: 4.7877, *p* < 0.01).

**TABLE 3:** Differential metabolites obtained from LC-MS metabolomics.

No	Name	Expected RT (min)	m/z (Expected)	Library score	p.Value	FDR	VIP	Fold change#	log2(FC)
1	Chenodeoxycholic acid/Ursodeoxycholic acid *neg	2.38	391.28538	71	0.0000118	.0014727	4.7877	15.47	3.9514
2	Homoveratric acid*neg	2.3	195.06628	100	0.0000833	.0025558	2.4907	15.15	3.9213
3	2,3-dihydroxybenzoate/3,4-dihydroxybenzoate *neg	0.7	153.01881	50	0.0000625	.0025377	3.259	9.5058	3.2488
4	P-Cresol sulfate*neg	.69	187.0065	51	0.0052808	.041315	1.3197	5.529	2.467
5	4-hydroxy-L-proline*pos	9.98	132.06604	50	0.00085038	.012062	1.7862	5.3485	2.4191
6	Trans-4-hydroxyproline*neg	10	130.05044	90	0.0001708	.0037896	2.1915	4.7831	2.2579
7	Hippurate*neg	5.73	178.05042	70	0.002848	.026427	1.4608	4.3439	2.119
8	2,6-Dimethylheptanoyl carnitine*pos	5.66	302.23261	57	0.0000399	.0022763	4.4995	4.0984	2.035
9	Trimethylamine N-oxide (TMAO)*pos	9.74	76.07624	50	0.00080664	.012062	1.8644	3.5867	1.8427
10	Indoxyl sulfate*neg	.77	212.00178	49	0.0000739	.0025377	2.7665	3.3896	1.7611
11	Decanoylcarnitine*pos	5.59	316.24824	85	0.00000858	.0014727	4.7877	3.3738	1.7544
12	4,8-Dimethylnonanoyl carnitine*pos	5.3	330.26391	54	0.0000221	.0014727	4.4995	3.3678	1.7518
13	4-Hydroxyindole*pos	.77	134.06005	43	0.00027296	.0051862	1.9629	3.2873	1.7169
14	Glycerylphosphorylethanolamine*pos	11.81	216.0637	50	0.00017096	.0037896	2.1053	2.9893	1.5798
15	3-Indoleacetic acid*neg	2.24	174.05605	60	0.0018614	.019133	1.5267	2.8326	1.5021
16	L-Octanoylcarnitine*pos	5.91	288.21696	64	0.0000763	.0025377	2.4986	2.7593	1.4643
17	Phosphorylcholine*pos	11.39	184.07332	70	0.0051518	.041315	1.333	2.612	1.3852
18	Glycerophosphocholine*pos	11.42	258.1101	70	0.0049662	.041281	1.3354	2.6038	1.3806
19	Dodecanoylcarnitine/Lauroylcarnitine *pos	5.41	344.27954	70	0.0018863	.019133	1.4789	2.5216	1.3344
20	LPE (18:2)-H*neg	5.91	476.27827	70	0.00082996	.012062	1.8604	2.4101	1.2691
21	LysoPE (18:1)*pos	5.86	480.30901	72	0.00011399	.0032486	2.4859	2.1129	1.0792
22	4-Pyridoxic acid*neg	1.2	182.04536	46	0.00096863	.012467	1.6082	.13182	-2.9233
22	4-Pyridoxic acid*pos	1.2	184.06096	40	0.001212	.014957	1.6081	.12098	-3.0471

Note: # represents the after-/before-treatment ratio. Among the metabolites with isomers, only one of them was listed in Figure 2.

In both models, only the level of 4-pyridoxinic acid decreased significantly (negative model, fold change: 0.13182, VIP: 1.6082, *p* < 0.01; positive model, fold change: 0.12098, VIP: 1.6081, *p* < 0.01). KEGG enrichment analysis revealed 10 pathways of enrichment, among which glycerophospholipid metabolism (hsa00564, *p* = 0.0005) and ether lipid metabolism (hsa00565, *p* = 0.0192) were significantly enriched (Table 4).

**TABLE 4:** Results of pathway analysis using MetaboAnalyst database.

No	Term	Description	Class	Total	Hits	*P*	Impact
1	hsa00564	Glycerophospholipid metabolism	Lipid metabolism	36	4	0.0005	0.2403
2	hsa00565	Ether lipid metabolism	Lipid metabolism	20	2	0.0192	0
3	hsa00330	Arginine and proline metabolism	Amino acid metabolism	38	2	0.0632	0.061
4	hsa00750	Vitamin B6 metabolism	Metabolism of cofactors and vitamins	9	1	0.0947	0
5	hsa00360	Phenylalanine metabolism	Amino acid metabolism	10	1	0.1047	0
6	hsa00592	alpha-Linolenic acid metabolism	Lipid metabolism	13	1	0.1341	0
7	hsa00561	Glycerolipid metabolism	Lipid metabolism	16	1	0.1625	0.0125
8	hsa04070	Phosphatidylinositol signaling system	Signal transduction	28	1	0.2677	0.0015
9	hsa00380	Tryptophan metabolism	Amino acid metabolism	41	1	0.3675	0
10	hsa00120	Primary bile acid biosynthesis	Lipid metabolism	46	1	0.4024	0

### 3.3 Construction of target-metabolic pathway network

**FIGURE 3:**
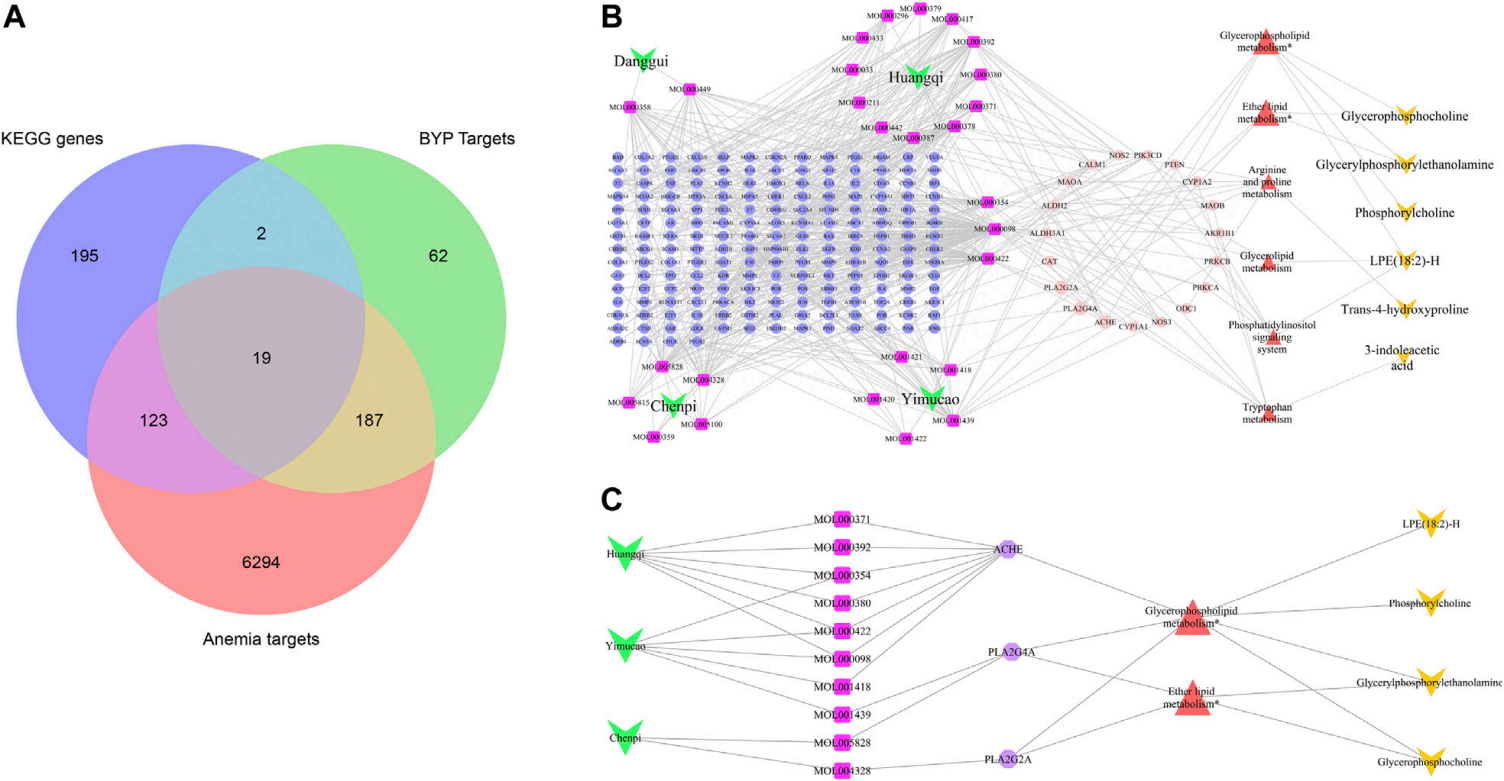
**(A)** Venn diagram of BYP targets, anemia targets and KEGG genes. **(B)** Active component–target–pathway interaction network (248 nodes, 602 edges). **(C)** Active component–corresponding target–significantly enriched metabolic pathway–potential biomarker interaction network (22 nodes, 34 edges). Green inverted triangle nodes represent the ingredients of BYP; pink square nodes represent the active molecules; round nodes represent potential targets (blue: targets not involved in pathways; orange: targets involved in pathways); red triangle nodes represent the metabolic pathways; orange circular nodes represent the targets of the metabolic pathways; yellow inverted triangle nodes represent the potential metabolites.

Thirty-two active components that met the screening criteria were extracted from the four main components of BYP by data mining (Table 5). We identified 270 BYP targets (drug targets), 6623 anaemia targets (disease targets), and 339 KEGG genes involved in the above 10 KEGG metabolic pathways. As presented in the Venn diagram (Figure 3A), BYP has 206 potential targets for anemia, of which 19 appear in the KEGG pathways (See Table 6 for details). To further demonstrate the relationship between the active components and affected metabolites and to reveal the anti-anaemia mechanism of BYP, an active components-targets-metabolic pathways-metabolomics network was constructed, which included six pathways and six metabolites (Figures 3B, C). Two significant enrichment pathways of glycerophospholipid metabolism and ether lipid metabolism were connected to the drug-target network through three targets: acetylcholinesterase (AchE), phospholipase A2 (PLA2) group IIA, and PLA2 group IVA.

**TABLE 5:** Main ingredients of Buxue Yimu Pills and their bioactive compounds.

Ingredients			Bioactive compounds			
Chinese pinyin name	English name	Latin name	Mol ID	Molecule name	OB (%)	DL
*Chenpi*	Dried Tangerine peel	*Citri Reticulatae Pericarpium*	MOL004328	naringenin	59.29	0.21
			MOL005100	(Rac)-Hesperetin	47.74	0.27
			MOL005815	Citromitin	86.9	0.51
			MOL005828	nobiletin	61.67	0.52
			MOL000359	sitosterol	36.91	0.75
*Danggui*	Angelica	*Angelicae Sinensis Radix*	MOL000358	beta-sitosterol	36.91	0.75
			MOL000449	Stigmasterol	43.83	0.76
*Huangqi*	Astragali Radix	*Hedysarum Multijugum Maxim*	MOL000392	formononetin	69.67	0.21
			MOL000422	kaempferol	41.88	0.24
			MOL000417	Calycosin	47.75	0.24
			MOL000438*	ZINC14758732	67.67	0.26
			MOL000098	quercetin	46.43	0.28
			MOL000239*	Jaranol	50.83	0.29
			MOL000398*	isoflavanone	109.99	0.3
			MOL000378	7-O-methy-lisomucronulatol	74.69	0.3
			MOL000354	isorhamnetin	49.6	0.31
			MOL000380	Astrapterocarpan	64.26	0.42
			MOL000371	3,9-di-O-methylnissolin	53.74	0.48
			MOL000442	1,7-Dihydroxy-3,9-dimethoxy pterocarpene	39.05	0.48
			MOL000439*	isomucronulatol-7,2′-di-O-glucosiole	49.28	0.62
			MOL000387	Bifendate	31.1	0.67
			MOL000374*	5′-hydroxyiso-muronulatol-2′,5′-di-O-glucoside	41.72	0.69
			MOL000433	FA	68.96	0.71
			MOL000296	hederagenin	36.91	0.75
			MOL000211	Mairin	55.38	0.78
			MOL000033	(24S)-24-Propylcholesta-5-ene-3beta-ol	36.23	0.78
			MOL000379	9,10-dimethoxypterocarpan-3-O-β-D-glucoside	36.74	0.92
*Yimucao*	Motherwort	*Leonurus cardiaca L*	MOL001439	arachidonic acid	45.57	0.2
			MOL000422	kaempferol	41.88	0.24
			MOL000098	quercetin	46.43	0.28
			MOL000354	isorhamnetin	49.6	0.31
			MOL001422	iso-preleoheterin	66.29	0.33
			MOL001421	preleoheterin	85.97	0.33
			MOL001418	galeopsin	61.02	0.38
			MOL001420	beta-Sitosterone	38	0.76

Note: * is used to represent the components not involved in the following network.

**TABLE 6:** Details of 19 potential candidate targets.

Gene symbol	Gene ID	Gene name
AchE	43	acetylcholinesterase (Cartwright blood group)
AKR1B1	231	aldo-keto reductase family 1 member B
ALDH2	217	aldehyde dehydrogenase 2 family (mitochondrial)
ALDH3A1	218	aldehyde dehydrogenase 3 family member A1
CALM1	801	calmodulin 1
CAT	847	catalase
CYP1A1	1543	cytochrome P450 family 1 subfamily A member 1
CYP1A2	1544	cytochrome P450 family 1 subfamily A member 2
MAOA	4128	monoamine oxidase A
MAOB	4129	monoamine oxidase B
NOS2	4843	nitric oxide synthase 2
NOS3	4846	nitric oxide synthase 3
ODC1	4953	ornithine decarboxylase 1
PIK3CD	5293	phosphatidylinositol-4,5-bisphosphate 3-kinase catalytic subunit delta
PLA2G2A	5320	phospholipase A2 group IIA
PLA2G4A	5321	phospholipase A2 group IVA
PRKCA	5578	protein kinase C alpha
PRKCB	5579	protein kinase C beta
PTEN	5728	phosphatase and tensin homolog

## 4 Discussion

### 4.1 Efficacy of BYP in anaemia and its possible influencing factors

After statistical analysis of the baseline characteristics and serological data of the patients in this study, it was found that the haemoglobin (*r* = –0.435, *p* = 0.003), haematocrit (*r* = –0.404, *p* = 0.007), and blood urea nitrogen levels (*r* = 0.454, *p* = 0.002), as well as patient age (*r* = 0.297, *p* = 0.045) and body mass index (*r* = 0.301, *p* = 0.042) before BYP treatment, were significantly correlated with the degree of anaemia improvement, with the first three parameters being moderately correlated and the last two being weakly correlated. Before and after BYP treatment, there were significant increases in haemoglobin, haematocrit, and red blood cells levels. Approximately 69.8% of patients who fully met the diagnosis of iron deficiency anaemia, that is, both iron deficiency (ferritin concentration threshold of 15 mg/L) and anaemia (haemoglobin concentration threshold of 120 g/L), showed no significant difference in the treatment outcome compared to those with non-iron deficiency anaemia.

### 4.2 Analysis of the effects of each component of BYP

In a study of 7682 anaemic Chinese herbal users and non-users, AR and ASR were found to be in the top 10 of the formula mono-herbs (Chen et al., 2018). According to Lipinski’s five principles, the active components of BYP were obtained (Shi et al., 2019). The main components of AR, such as polysaccharides, flavonoids, and AS-IV, have been studied and confirmed to promote erythrocyte differentiation, and increase γ-globin mRNA expression and foetal haemoglobin synthesis (Shi et al., 2019). It has unique haematopoietic function, anti-inflammatory activity, and immune properties. The main components of ASR are stigmasterol, ferulic acid, volatile oil, and other phthalate esters containing phthalic acid mother nuclei (Wei et al., 2016). In animal models of iron deficiency anaemia, ASR has been observed to significantly inhibit the expression of ferritin by blocking the JAK-STAT, BMP-Smad, and ERK pathways (Zhang et al., 2014). LC has been used for more than 1,800 years to improve menstrual blood stasis and delivery disorders, including dysmenorrhoea, amenorrhoea, and postpartum haemorrhage (Miao et al., 2019). Its total alkaloid extract and major components, including stachydrine, have been shown to significantly reduce interleukin-1β-induced NO, PGE2, interleukin-6, and tumour necrosis factor (TNF)-α production by inhibiting the activation of the PI3K/Akt/NF-κB signalling pathway (Chen, 2021). CRP is also used in several well-known TCM prescriptions for the treatment of blood stasis syndrome (Yu et al., 2018). Previous studies on the overall pharmacodynamics of TCM have demonstrated that BYP components can partially support its therapeutic potential for angiogenic defects and haemodynamic disorders (Zhang et al., 2022).

According to our network pharmacological identification, kaempferol (MOL000422, the only given ID from TCMSP), quercetin (MOL000098), and isorhamnetin (MOL000354) are three components commonly found in AR and LC. After network analysis using Cytoscape, quercetin (MOL000098) was identified as the most important compound, which may reduce oxidative stress, improve iron status, and protect red blood cells, thus improving inflammation and chemotherapy-related anaemia.

### 4.3 Metabolic pathway analysis

To elucidate the mechanism of TCM in the treatment of anaemia, previous studies have focused on animal experiments. In this experiment, in women of childbearing age, a combination of metabolomics and network analysis was used to obtain significantly enriched metabolic pathways, with glycerophospholipid, and ether lipid metabolism as the top two pathways associated with anaemia and BYP efficacy. Glycerophospholipid metabolism plays a role in maintaining normal cellular function and enhancing lipid metabolism, thereby stabilising cell membranes. Ether lipid metabolism is less frequently reported to be directly related to correcting anemia, but it has been considered to have an important role in platelet function (Chignard et al., 1985) and reducing cholestasis (Chen et al., 2016), and therefore may contribute to improvement of anemia indirectly. Previously, systematic studies on Danggui Buxue decoction, which is composed of ASR and AR, proposed three metabolic disorder pathways involving iron ion binding, haematopoiesis, reactive oxygen species production, inflammation, and apoptosis (Liu et al., 2021).

In our study, a human metabolic profile was set where the major differential metabolites were lipid derivatives, and half of the metabolic pathways (5/10) were categorised as lipid metabolism. These significantly altered metabolites were also found to have several therapeutic properties, including the inhibition of inflammation and oxidative stress. Chenodeoxycholic acid was the most significantly increased metabolite and thought to have many beneficial functions, including alleviation of cholestasis, antiinflammatory effects, and modulation of immune function (Chappell et al., 2018). Lang et al. found that cholestasis accelerates reduction of circulating red blood cells, leading to anaemia (Lang et al., 2016). 4-pyridoxycholic acid was the only differential metabolite with reduced concentrations, which may be related to the fact that it is a catabolic product of vitamin B6, a cofactor of many important enzymes involved in a range of biochemical reactions, including haemoglobin synthesis. Reinken and Kurz demonstrated that as haeme synthesis improved, the requirement for pyridoxal phosphate increased, and the vitamin B6 body pool decreased (Reinken and Kurz, 1978), which are consistent with our findings.

### 4.4 Target prediction

Using a combination of network analysis and metabolomics, we identified 19 candidate targets, of which the most potential therapeutic targets were AchE, PLA2 group IIA, and PLA2 group IVA. The enzymatic activity of erythrocyte membrane AchE has been found to be a biomarker of membrane integrity (normal), senescence (low), inflammation (high), and neurotoxicity (high) (Saldanha, 2017). Xu et al. experimentally found that EPO-induced transcription and expression of α- and β-bead proteins can only be significantly enhanced when AchE is overexpressed. AchE may regulate the responsiveness of erythroid-like cells to EPO, and one hypothesis is that AchE assists in stabilising EPO receptors *via* RACK1 in lipid rafts, resulting in better sensitisation signals with important implications for erythropoiesis and adult erythroid maturation (Xu et al., 2019). In addition, BYP may also act by inhibiting the activity of important isoforms of PLA2 IIA and IVA. The PLA2 family catalyses the release of arachidonic acid and others from membrane phospholipids, which are then metabolised by COX and LOX to produce eicosanoids that trigger inflammatory responses and play a central role in cellular lipid metabolism and signal transduction (Tan and Goh, 2017; Mouchlis and Dennis, 2019). Group IIA sPLA2 often acts as an amplifier of pathological inflammation (Scott et al., 2021). Group IVA cPLA2 gene is located in the 1q25 region of the human chromosome and causes an upstream of several proinflammatory mediators (Yarla et al., 2016). These two proteins can activate each other through MAPK cascade reactions; thus, BYP may potentially improve anaemia by regulating intracellular signalling pathways, inhibiting IIA-dependent production of second messengers such as PGE2, reducing Ca2+ channel activation, inhibiting inflammatory responses and platelet activation, stabilising metabolism, avoiding coagulation problems such as excessive thrombosis, and reducing microcirculatory disorders. However, we must not overlook the fact that moderate platelet activation may play a role in enhancing haemostasis, which involves complex platelet activation signalling pathways that need to be further studied. It has also been found that ferritin has a strong pro-oxidant effect and is considered a potentiometric modulator of plasma LP-PLA2 activity and a good inducer of elevated TNF-α (Ragab et al., 2015). Oral iron supplementation often leads to the elevation of ferritin, which may promote a series of responses mediated by inflammatory factors such as PLA2 or TNF-α. Otherwise, BYP, owing to unaltered iron metabolism, may avoid the above, improving anaemia.

BYP has unique advantages over other forms of anaemia treatments. First, it does not affect iron metabolism but rather affects iron distribution. Haematopoiesis increases, and iron from the non-haematopoietic system (blood circulation and iron storage cells) can be transported more efficiently to the haematopoietic system (red lineage precursors), thus optimising the redistribution of iron. Second, after one course of BYP treatment, there was no significant change in haematocrit and reticulocyte count, nor was there any sign of elevated arterial blood pressure, probably because BYP promotes haematopoiesis but does not trigger adverse erythropoietic and haemodynamic effects; however, further studies are needed to confirm this result. Third, inflammatory processes can interfere with ideal iron regulation, increasing circulating hepcidin and limiting iron transport to red blood cells, thereby causing or exacerbating anemia (Benson et al., 2021). Chronic inflammation can lead to imbalances in the production of haematopoietic stem cells in the blood and cause an impairment in the self-renewal of haematopoietic stem cells and red blood cell production. In contrast, BYP inhibits inflammation and apoptosis, activates repair, and may reduce serum hepcidin levels, improve anaemia, and have adjuvant effects in improving oxidative stress and its related diseases.

## 5 Conclusion

BYP can improve anaemia without altering the iron metabolism. Since BYP is a mixed prescription of TCM, it is hypothesised that its therapeutic effect may be a combination of multiple components and targets. After treatment, 22 differential metabolites were identified, which were mainly related to the inhibition of inflammation and oxidative stress. Combined network analysis and metabolomics revealed that AchE and PLA2 groups IIA and IVA might be the most promising therapeutic targets for promoting haematopoiesis. The combination of network analysis and metabolomics complements each other and provides a three-dimensional approach to TCM research.

## Data Availability

The raw data supporting the conclusions of this article will be made available by the authors, without undue reservation.

## Abbreviations

ACC, Asini Corii Colla; AchE, acetylcholinesterase; AR, Astragali Radix; ASR, Angelicae Sinensis Radix; BYP, Buxue Yimu pills; CRP, Citri Reticulatae Pericarpium; EPO, erythropoietin; KEGG, Kyoto Encyclopedia of Genes and Genomes; LC, Leonurus cardiaca L.; LC-MS, liquid chromatography mass spectrometry; MS, mass spectrometry; OPLSDA, orthogonal partial least squares discriminant analysis; PLA2, phospholipase A2; PCA, principal component analysis; PLSDA, partial least squares discriminant analysis; TCM, traditional Chinese medicine; TNF, tumour necrosis factor; VIP, variable importance.
