# Supplementary material for: Metabolomic mechanism and pharmacodynamic material basis of Buxue Yimu pills in the treatment of anaemia in women of reproductive age
Source: Front Pharmacol. 2023 Jan 10;13:962850. doi: 10.3389/fphar.2022.962850 (PMC9871362; doi:10.3389/fphar.2022.962850)
Supplement: Supplementary file 1 [file DataSheet2.pdf]

Instructions for Buxue Yimu Pills

Please read the instructions carefully and use them under the guidance of physicians

Pregnant women banned

【Name of Drug】

General name: Buxue Yimu Pills

Chinese Pinyin: Buxue Yimu Wan

【Composition】 Danggui, Huangqi, Ejiao, Yimucao, Chenpi

【Description】 This product is a brown concentrated pill; fragrant, bitter in taste.

【Indications】 To supplement Qi and Blood, remove blood stasis.

【Specification】 12g per bag (12g per 200 pills)

【Dosage and Administration】 Orally.12g once, twice a day.

【Adverse Reactions】 Not yet clear

【Contraindications】 This product should not used in pregnant women.

【Precautions】 Avoid raw, cold, and spicy food.

【Storage】 Seal it and put it in a cool and dry place.

【Package】 Pharmaceutical packaging composite film.10 Bags per box.

【Expiry】 18 months.

【Approval No.】 CFDA No. Z20090602

【Spercification No.】 CFDA Standard No. YBZ083112009

【Manufacturer】

Name: Zhuzhou Qianjin Pharmaceutical Co., Ltd.

Address: Jinhook Mountain Road, Hetang District, Zhuzhou City, Hunan Province

Postal code: 412003

Telephone number.: Marketing service: 0731-5777199 800-8786502

Quality medication: 0733-2490521 800-8788996

Fax number: 0733-2493188

Registered address: Building 13, Commercial Street, Yandi Square, Zhuzhou High-tech Development Zone

Website: http://www.qian-jin.com

Notes: This is not an official version, for your information only. If you have any question, please contact the authors.

核准日期：　年　月　日

补血益母丸说明书

请仔细阅读说明书并在医师指导下使用

孕妇禁服

【药品名称】

通用名称：补血益母丸

汉语拼音：Buxue　Yimu　Wan

【成　　份】当归、黄芪、阿胶、益母草、陈皮。

【性　　状】本品为棕褐色的浓缩丸；气香,味苦。

【功能主治】补益气血，祛瘀生新。用于气血两虚兼血瘀证产后腹痛。

【规　　格】每袋装 12g（每 200 丸重 12g）

【用法用量】口服。一次 12g，一日 2 次。

【不良反应】尚不明确。

【禁　　忌】孕妇禁服。

【注意事项】忌生冷辛辣。

【贮　　藏】密封，置阴凉干燥处。

【包　　装】 复合膜包装。10 袋/盒。

【有 效 期】18 个月。

【批准文号】

【生产企业】

企业名称：株洲千金药业股份有限公司

生产地址：湖南省株洲市荷塘区金钩山路

邮政编码：412003

电话号码：营销服务咨询：0731-5777199　　800-8786502

质量用药咨询：0733-2490521　　800-8788996

传真号码：0733-2493188

注册地址：株洲高新技术开发区炎帝广场商业街 13 栋

网　　址：http：//www.qian-jin.com
