# Supplementary material for: Metabolomic mechanism and pharmacodynamic material basis of Buxue Yimu pills in the treatment of anaemia in women of reproductive age
Source: Front Pharmacol. 2023 Jan 10;13:962850. doi: 10.3389/fphar.2022.962850 (PMC9871362; doi:10.3389/fphar.2022.962850)
Supplement: Supplementary file 2 [file DataSheet1.PDF]

4-hydroxy-L-proline

Positive mode

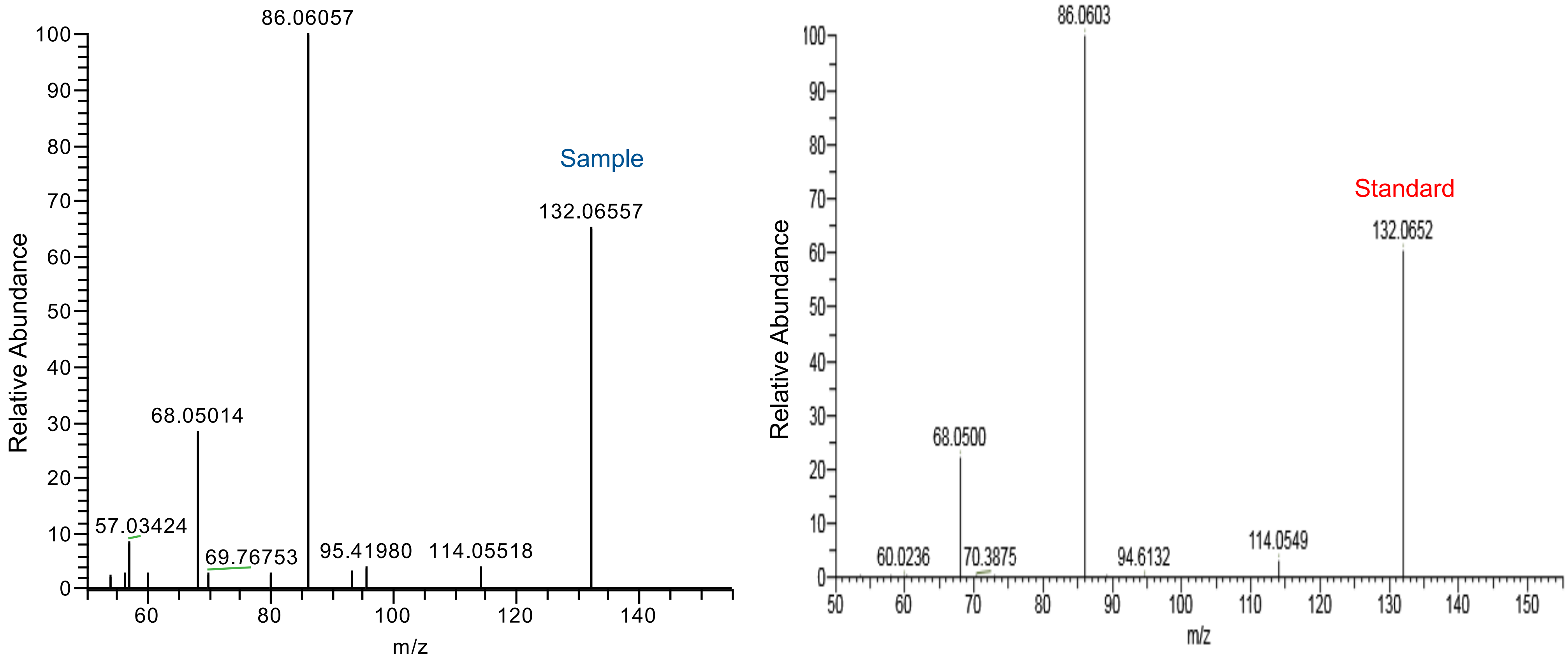

Supplementary Fig. 1-1: The secondary mass spectrogram of 4-hydroxy-L-proline. Left: sample; right: standard.

2,6-dimethylheptanoyl carnitine

Positive mode

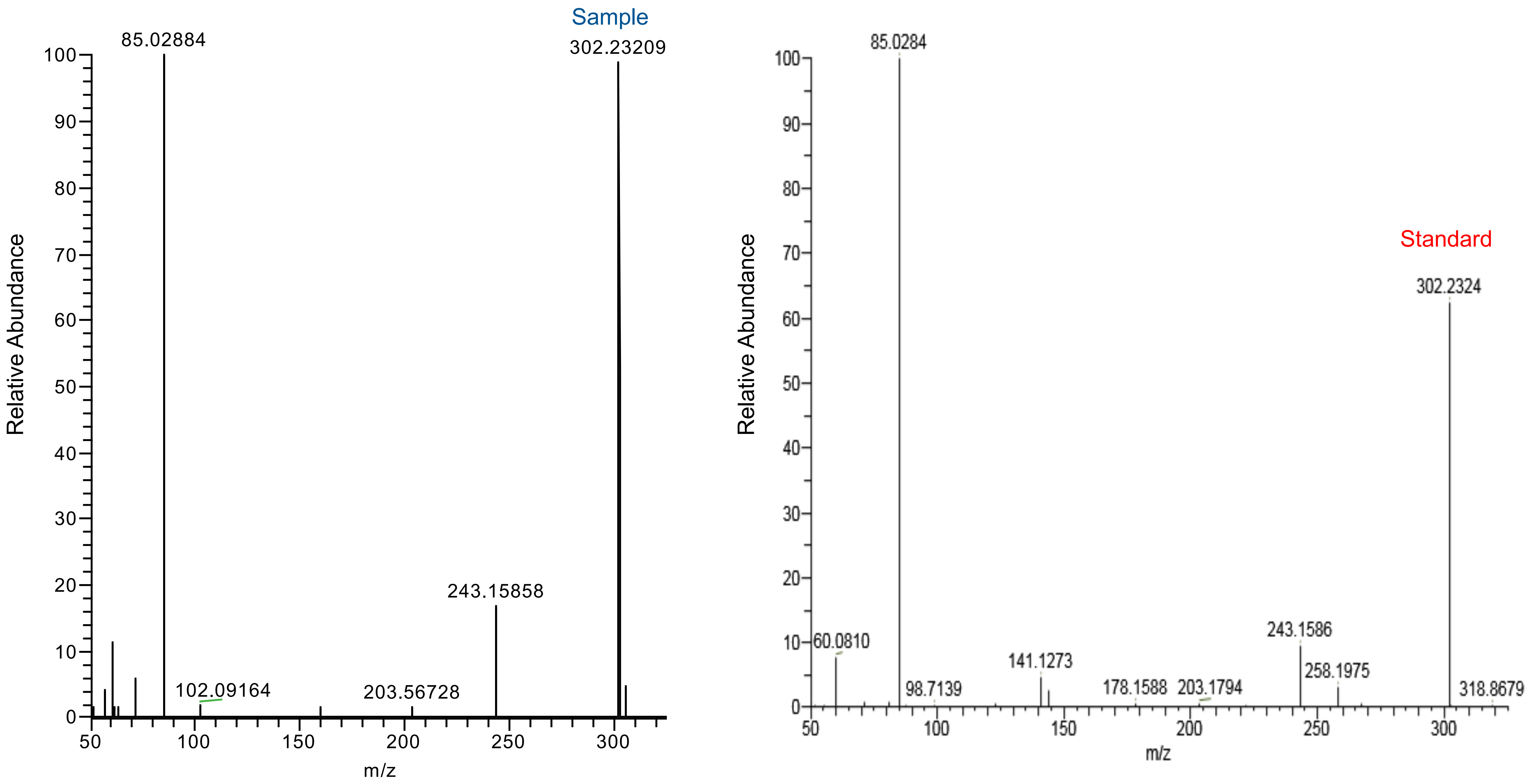

Supplementary Fig. 1-2: The secondary mass spectrogram of 2,6-dimethylheptanoyl carnitine. Left: sample; right: standard.

Trimethylamine N-oxide(TMAO)

Positive mode

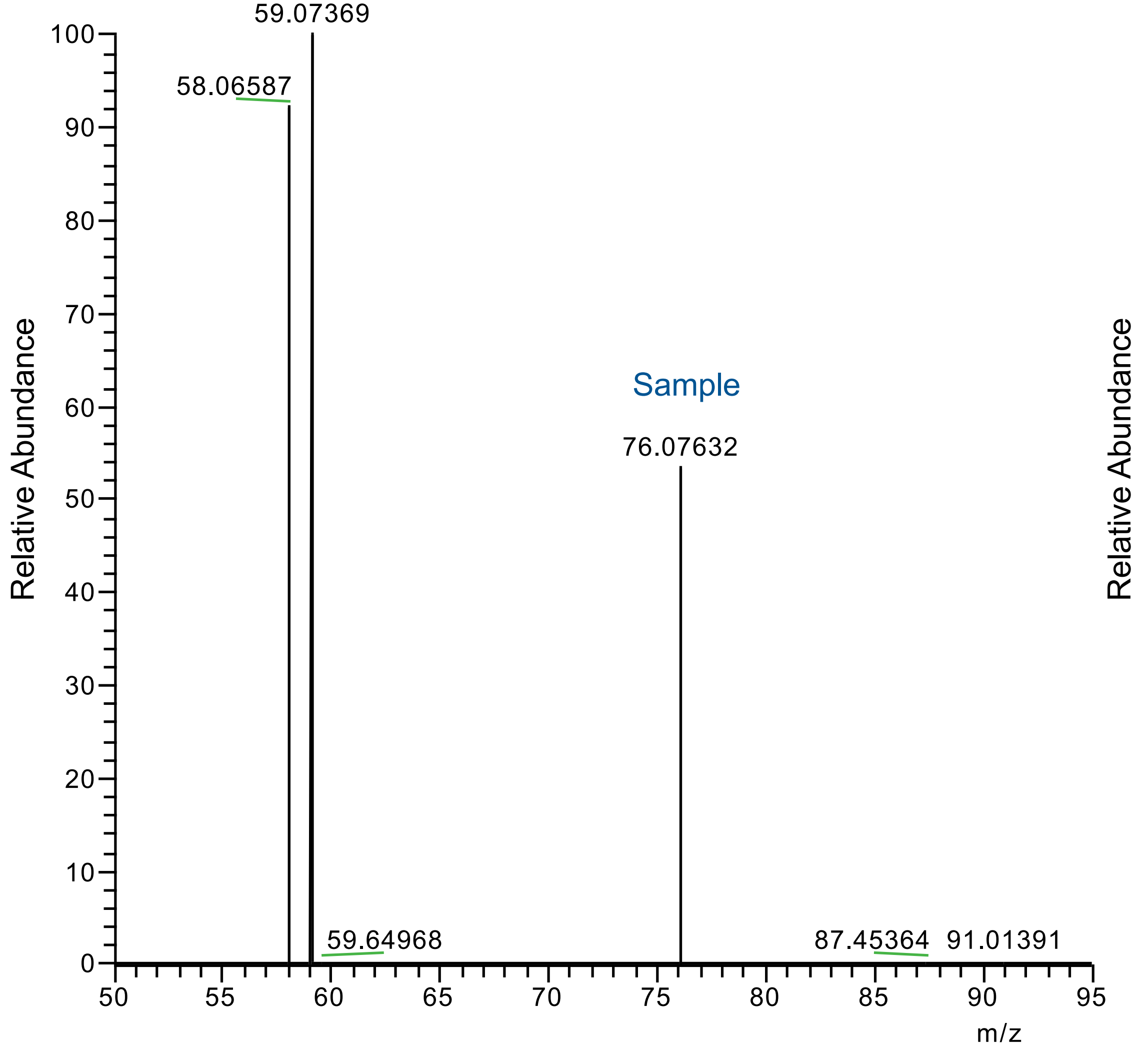

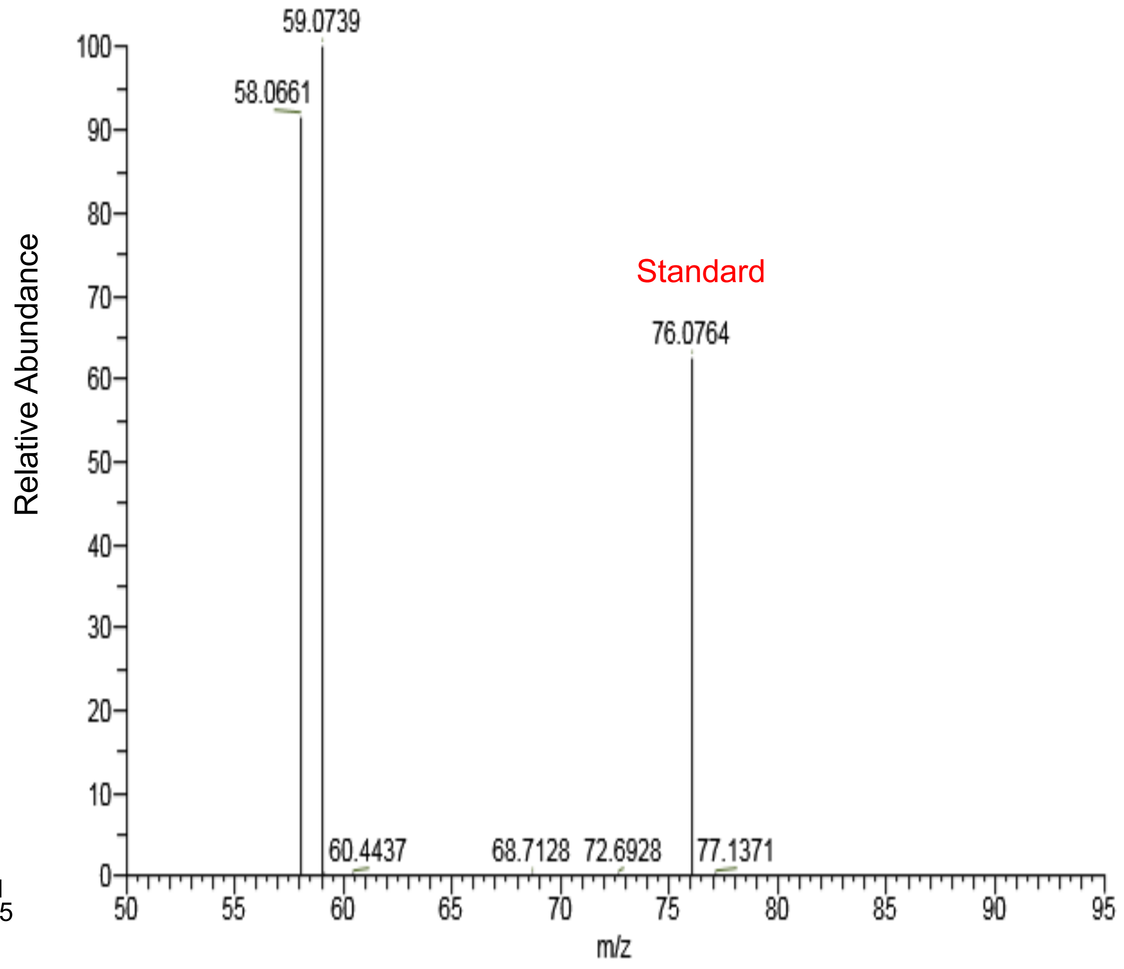

Supplementary Fig. 1-3: The secondary mass spectrogram of trimethylamine N-oxide(TMAO). Left: sample; right: standard.

Decanoylcarnitine

Positive mode

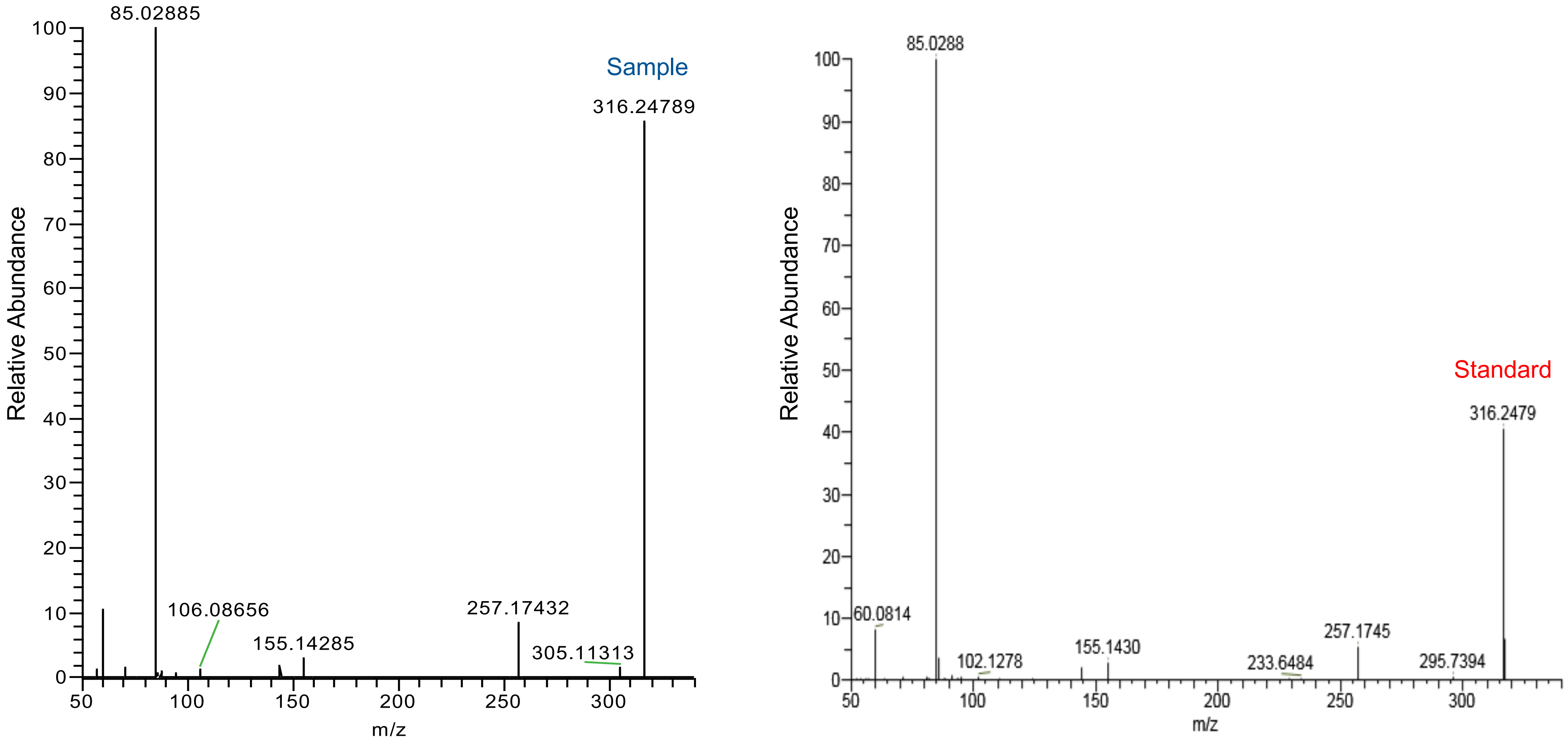

Supplementary Fig. 1-4: The secondary mass spectrogram of decanoylcarnitine. Left: sample; right: standard.

4,8-dimethylnonanoyl carnitine

Positive mode

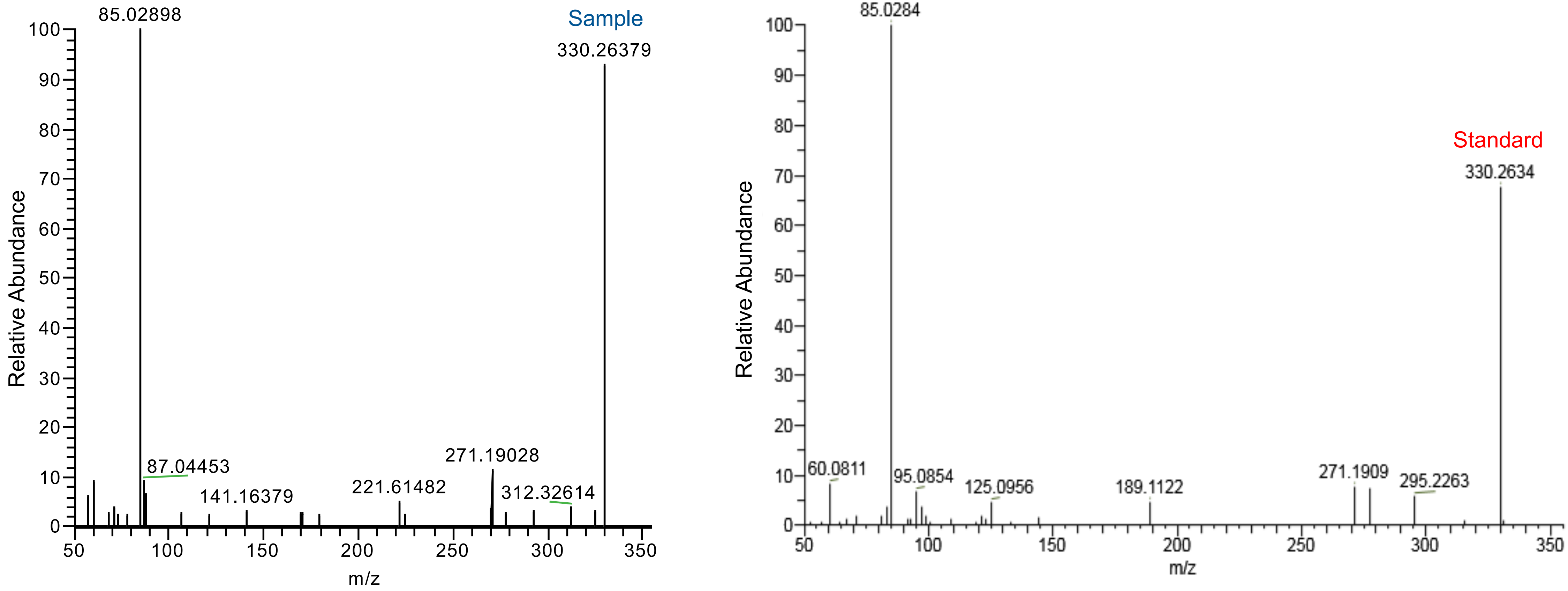

Supplementary Fig. 1-5: The secondary mass spectrogram of 4,8-dimethylnonanoyl carnitine . Left: sample; right: standard.

4-hydroxyindole

Positive mode

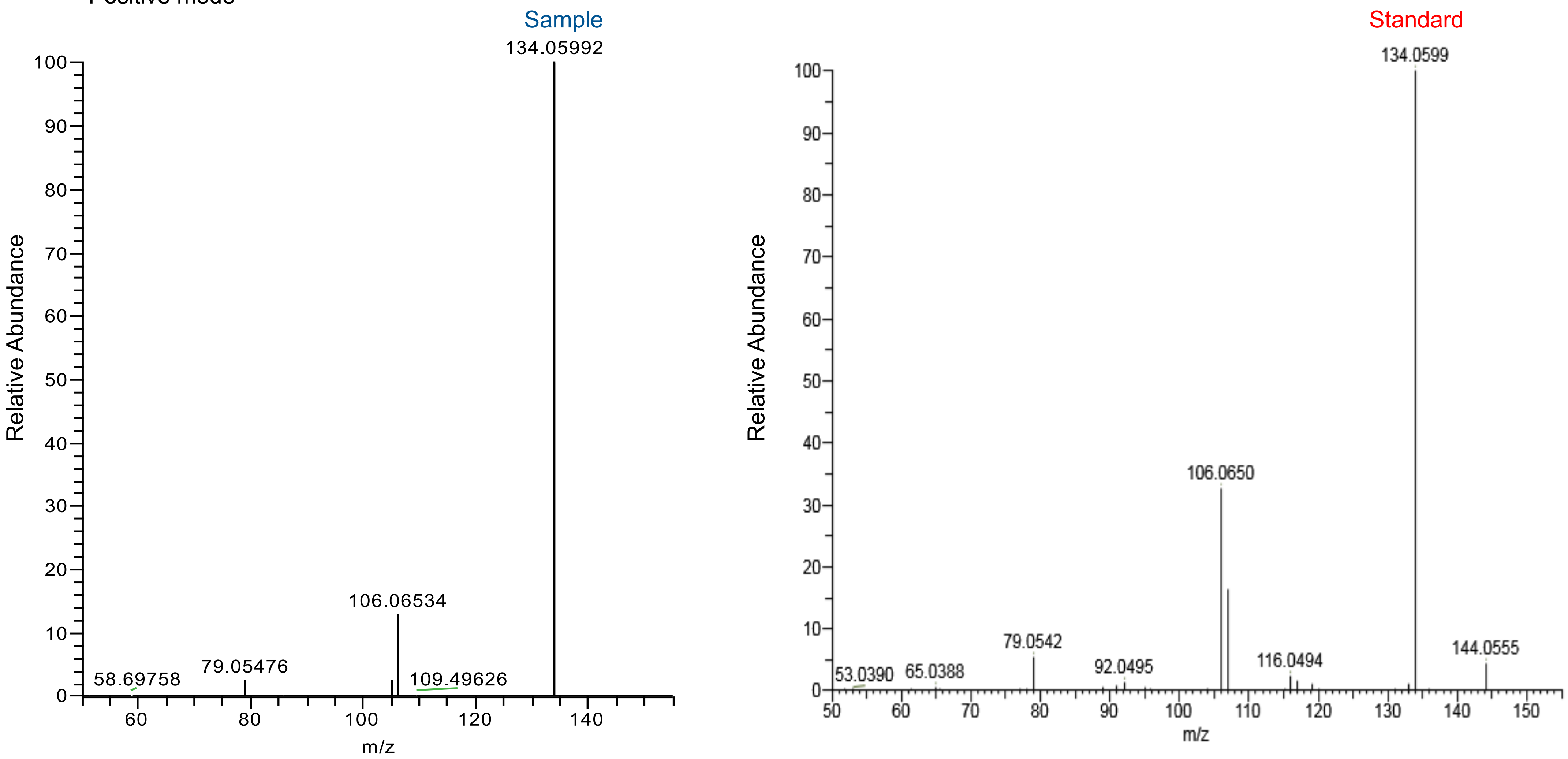

Supplementary Fig. 1-6: The secondary mass spectrogram of 4-hydroxyindole. Left: sample; right: standard.

Glycerylphosphorylethanolamine

Positive mode

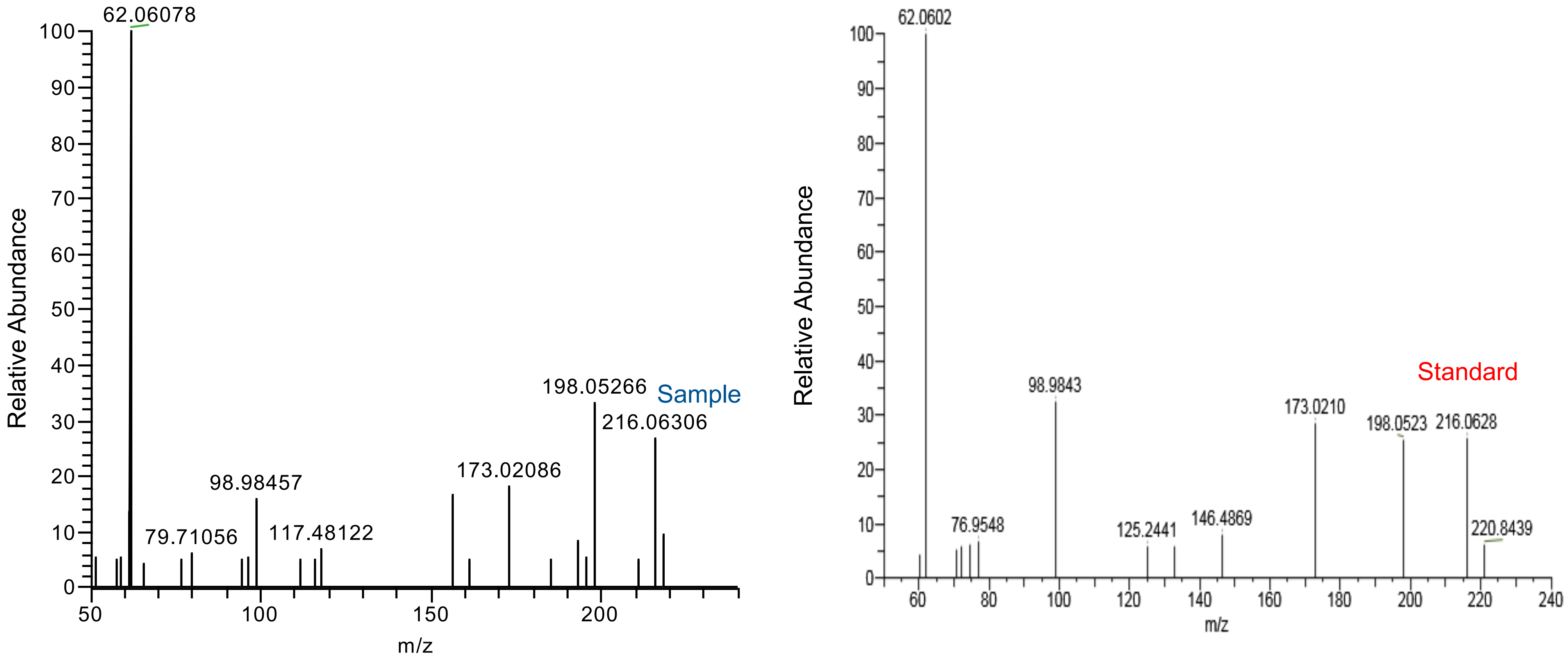

Supplementary Fig. 1-7: The secondary mass spectrogram of glycerylphosphorylethanolamine. Left: sample; right: standard.

L-octanoylcarnitine

Positive mode

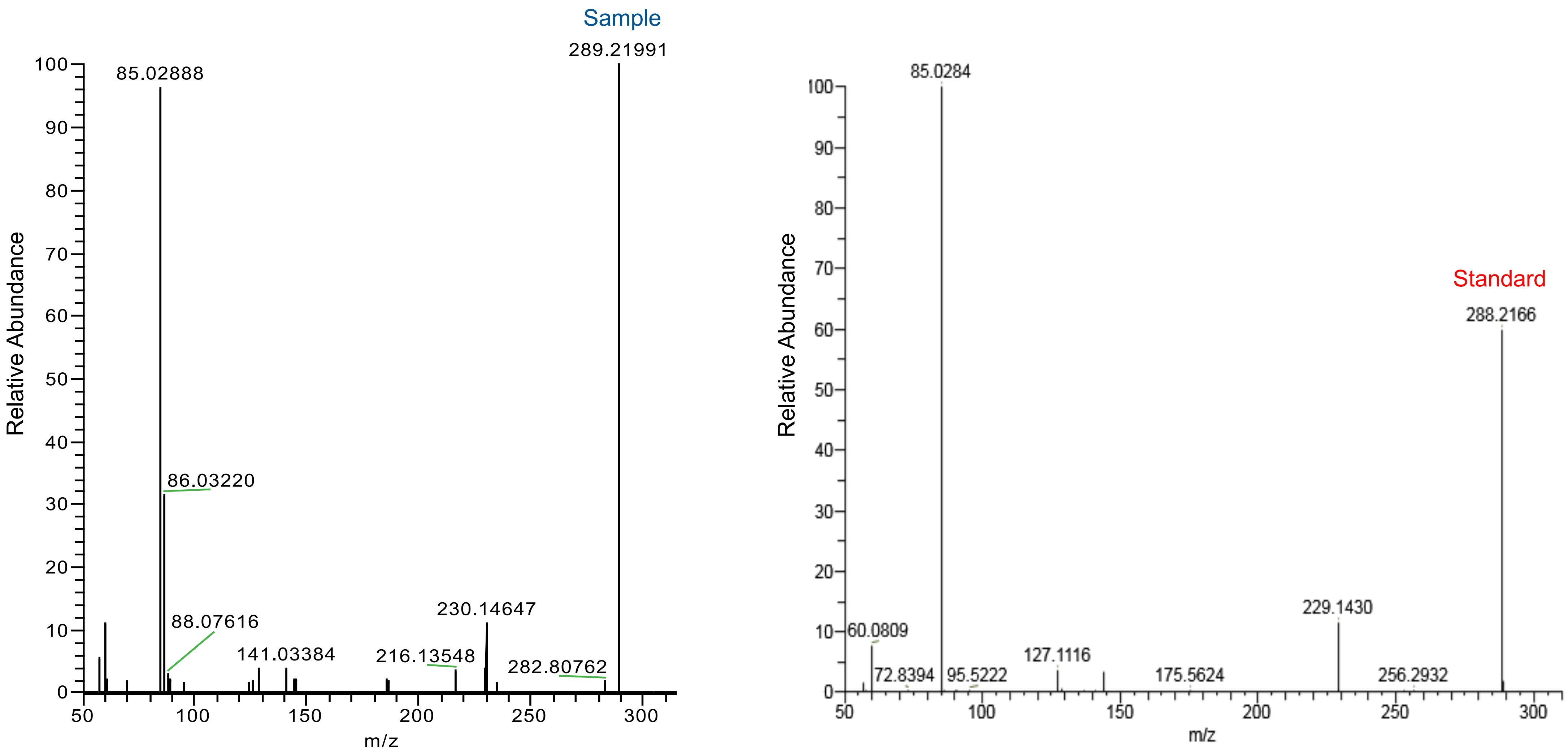

Supplementary Fig. 1-8: The secondary mass spectrogram of l-octanoylcarnitine. Left: sample; right: standard.

Phosphorylcholine

Positive mode

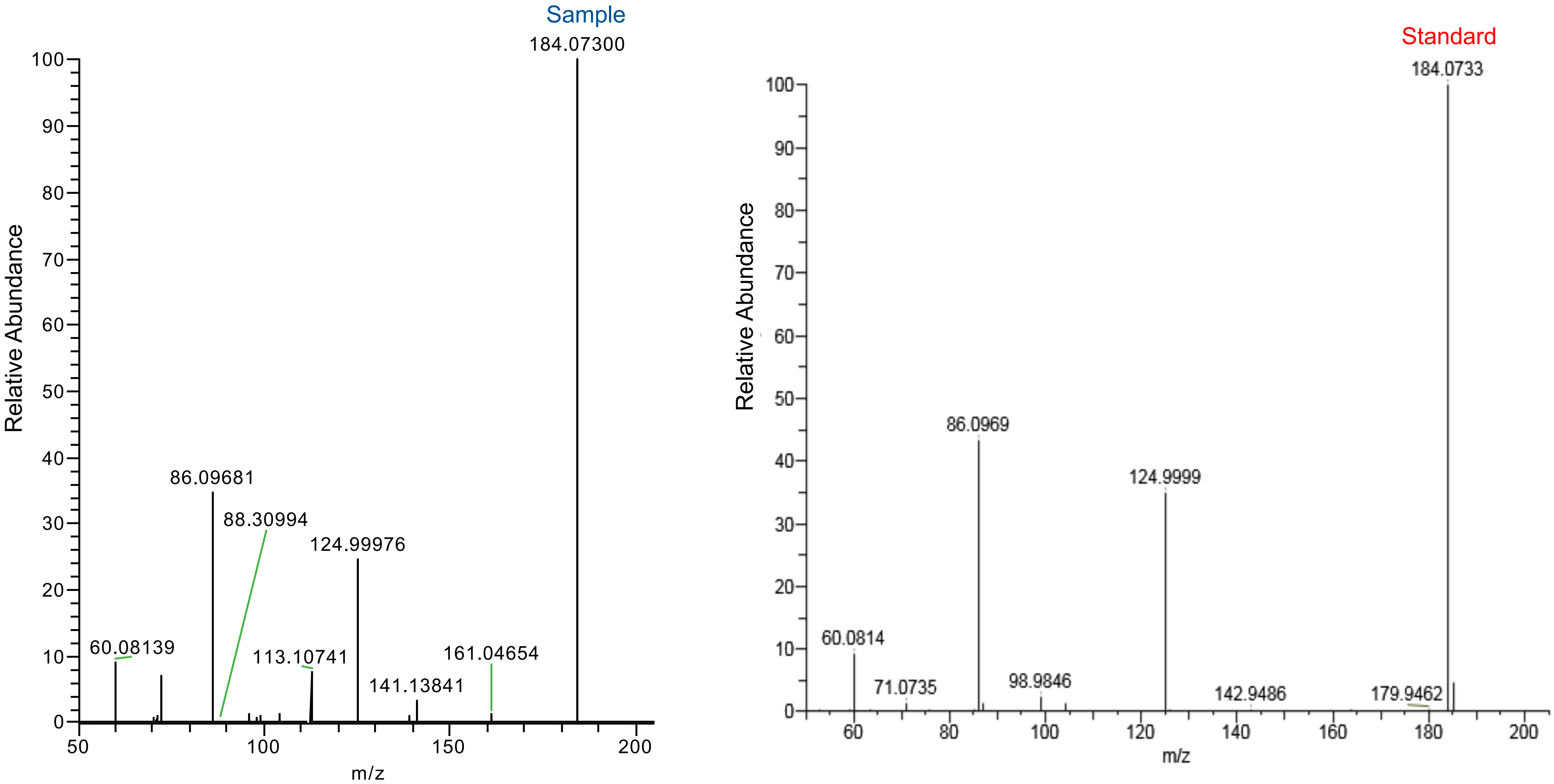

Supplementary Fig. 1-9: The secondary mass spectrogram of phosphorylcholine. Left: sample; right: standard.

Glycerophosphocholine

Positive mode

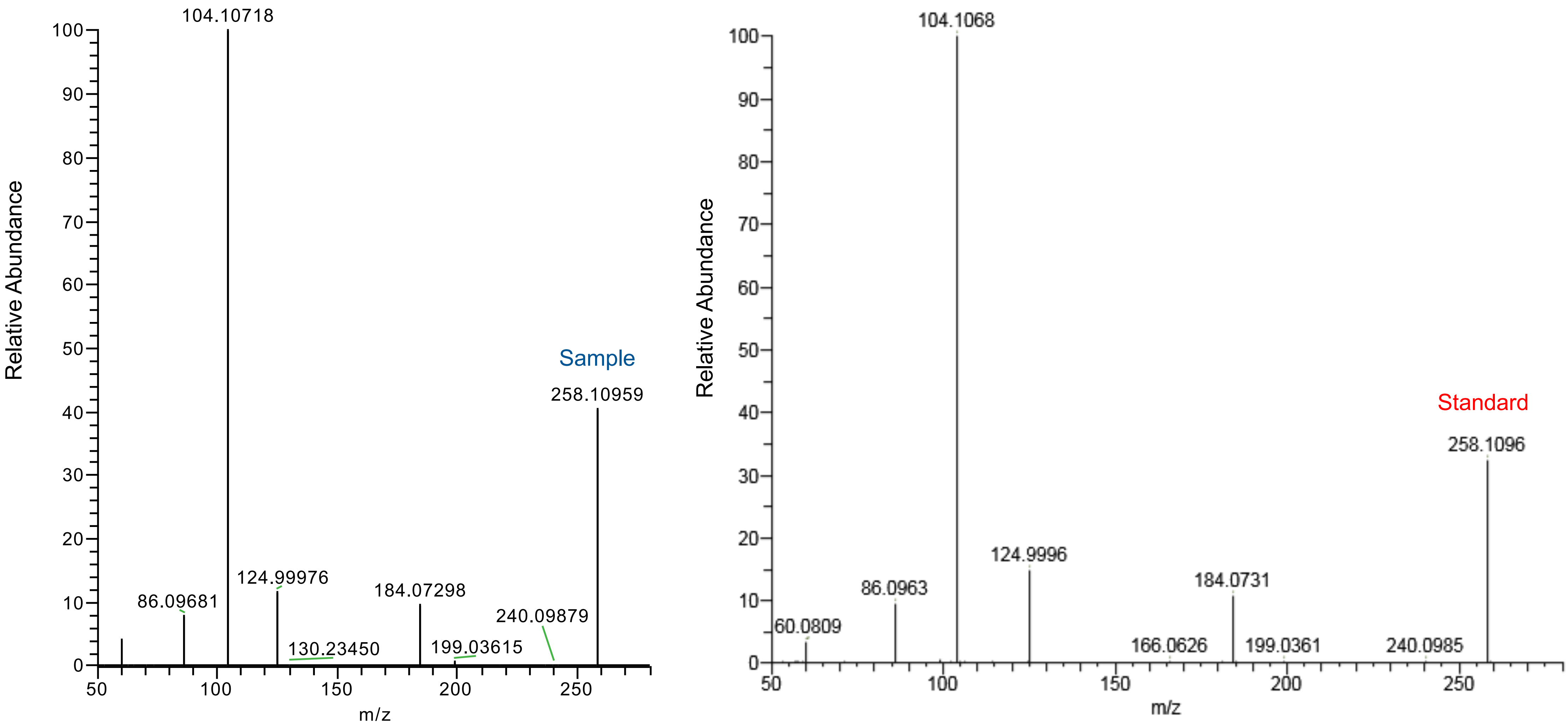

Supplementary Fig. 1-10: The secondary mass spectrogram of glycerophosphocholine. Left: sample; right: standard.

Dodecanoylcarnitine/lauroylcarnitine

Positive mode

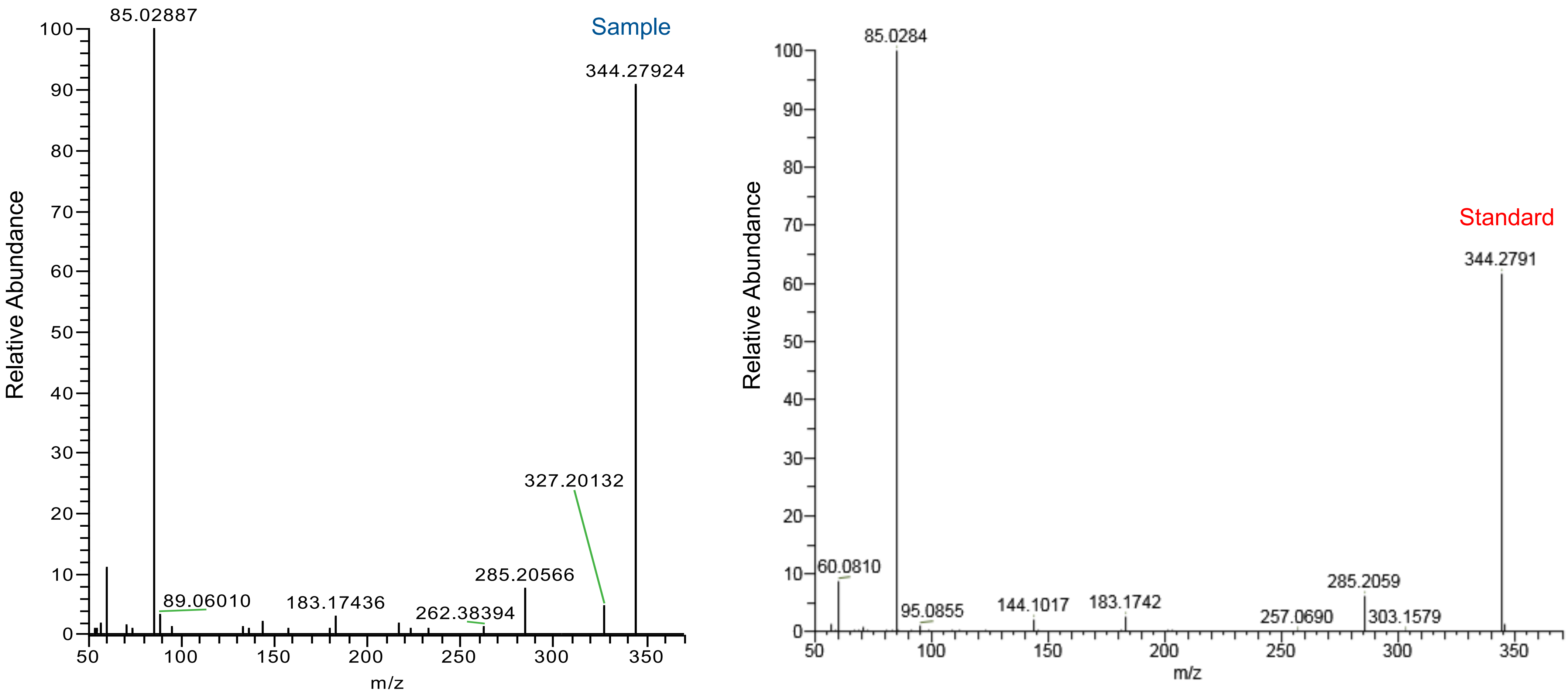

Supplementary Fig. 1-11: The secondary mass spectrogram of dodecanoylcarnitine/lauroylcarnitine. Left: sample; right: standard.

LysoPE(18:1)

Positive mode

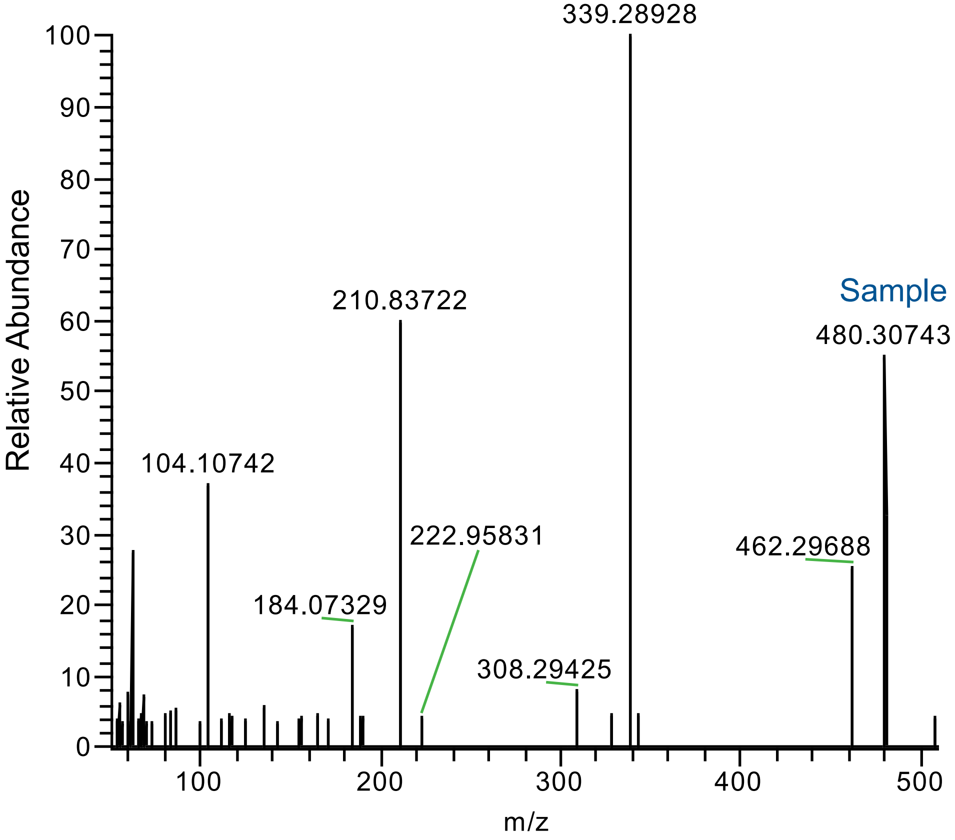

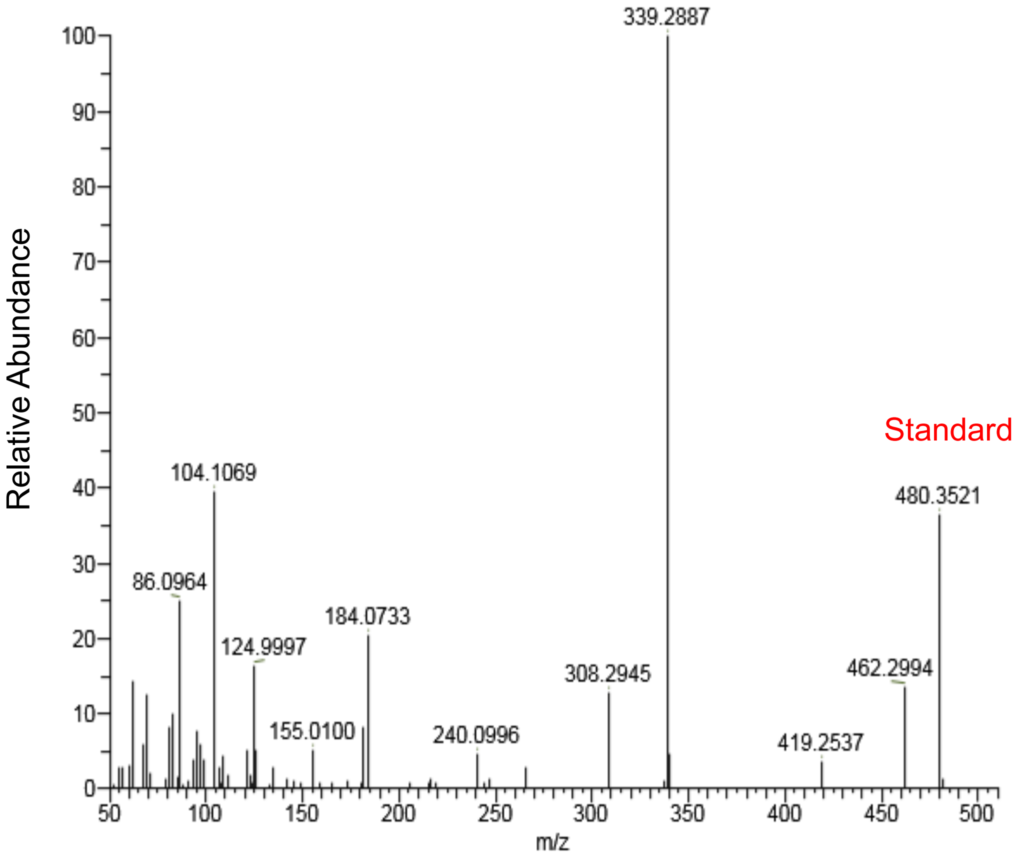

Supplementary Fig. 1-12: The secondary mass spectrogram of LysoPE(18:1) . Left: sample; right: standard.

4-pyridoxic acid

Positive mode

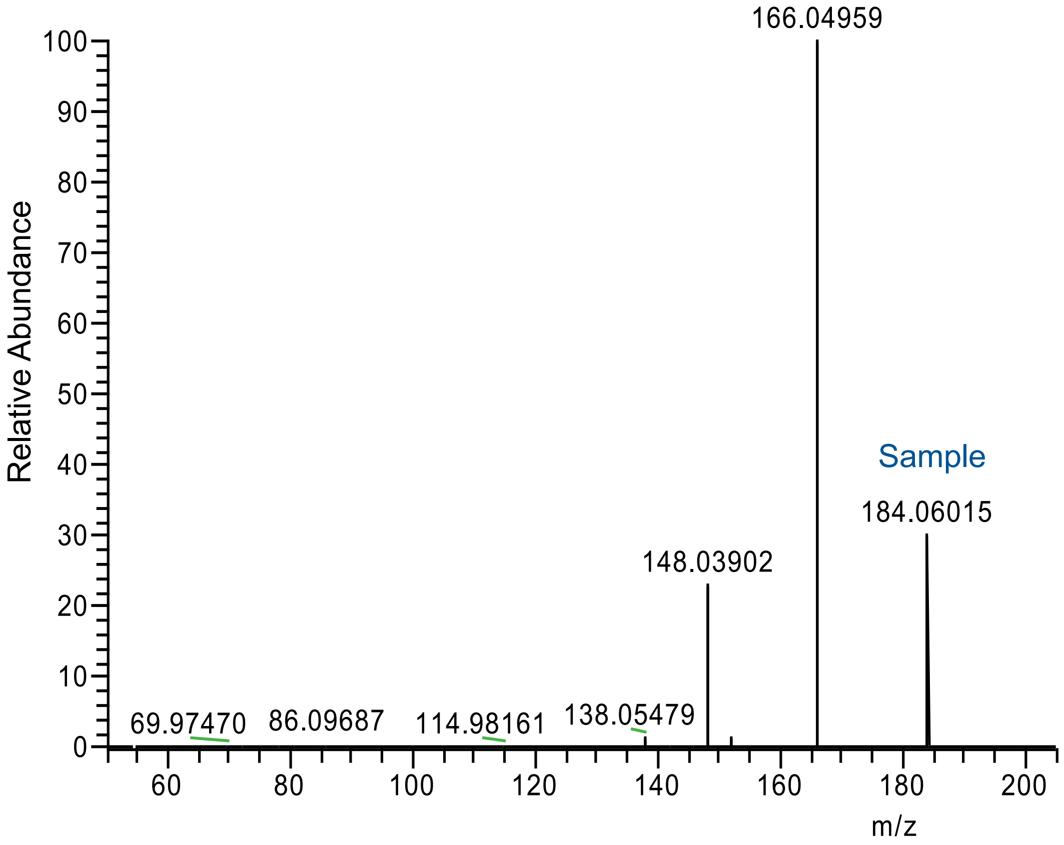

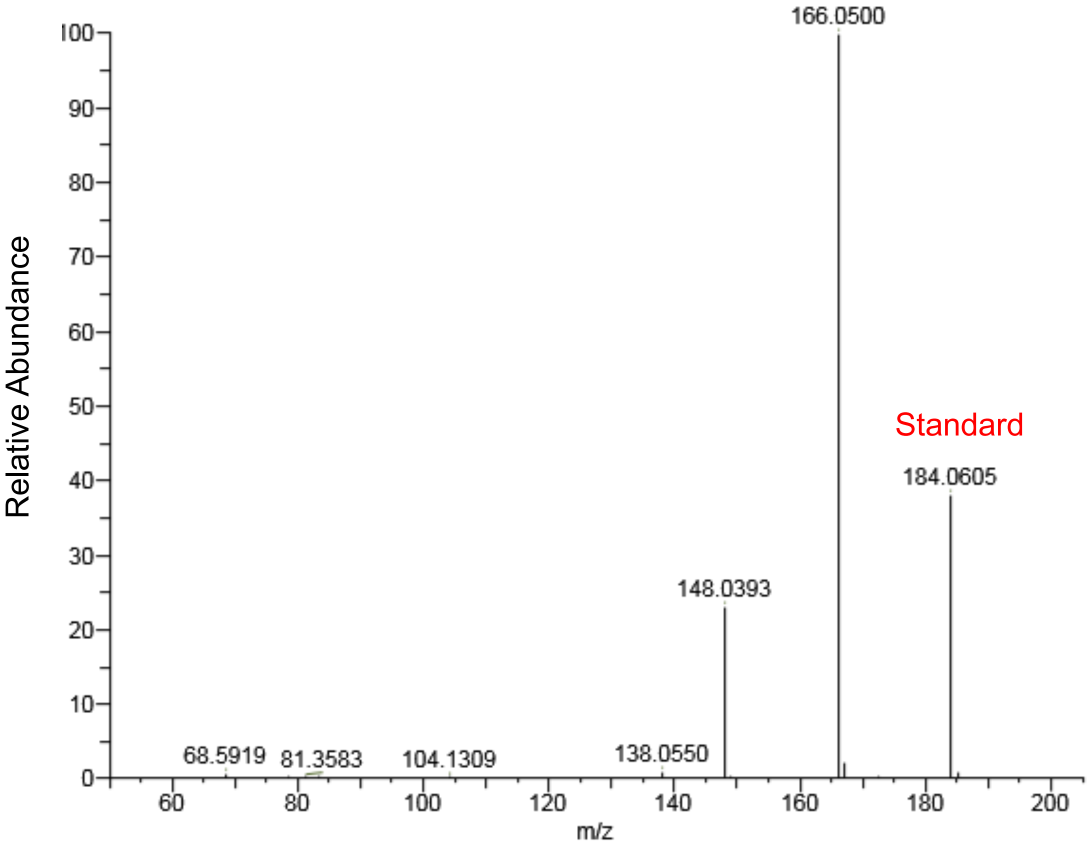

Supplementary Fig. 1-13: The secondary mass spectrogram of 4-pyridoxic acid. Left: sample; right: standard.

Chenodeoxycholic acid/ursodeoxycholic acid

Negative mode

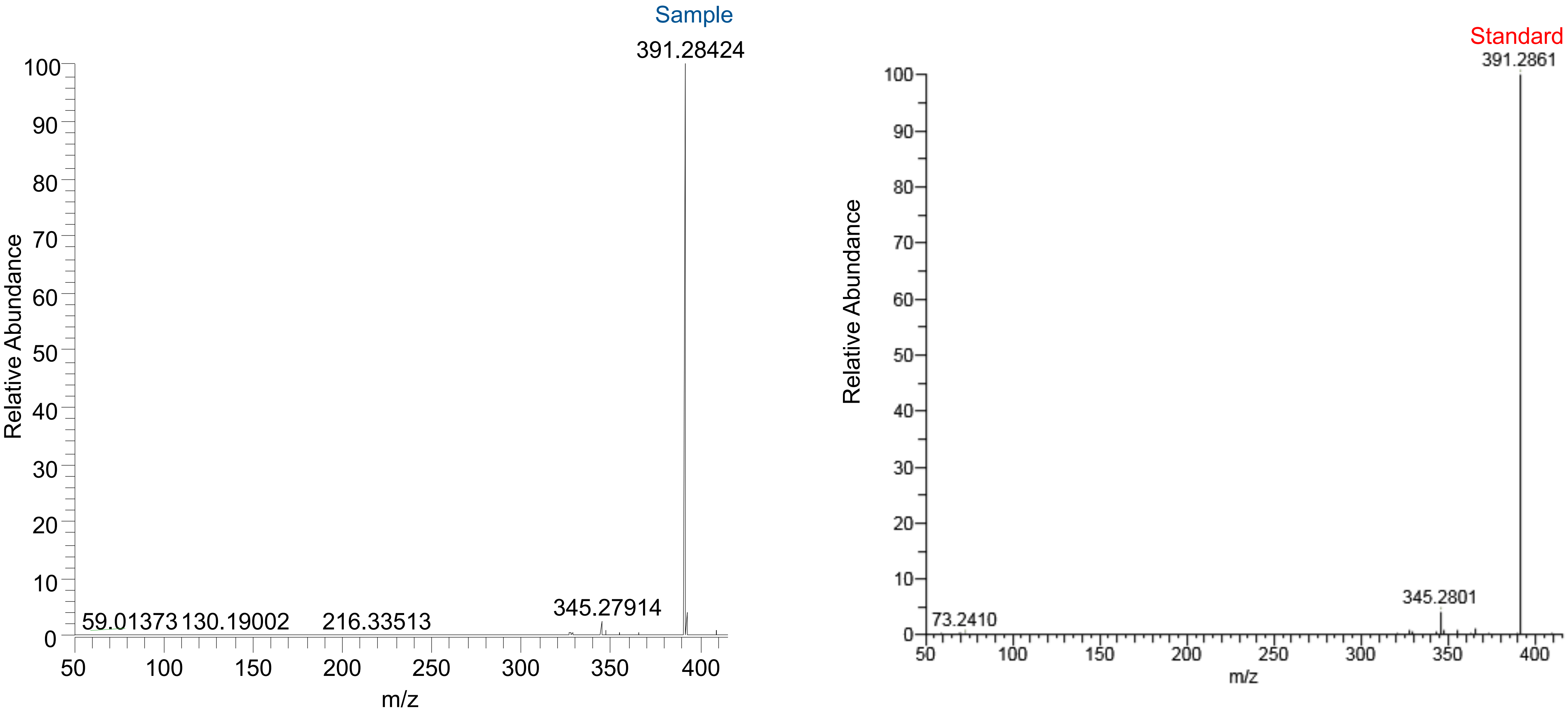

Supplementary Fig. 1-14: The secondary mass spectrogram of chenodeoxycholic acid/ursodeoxycholic acid. Left: sample; right: standard.

Homoveratric acid

Negative mode

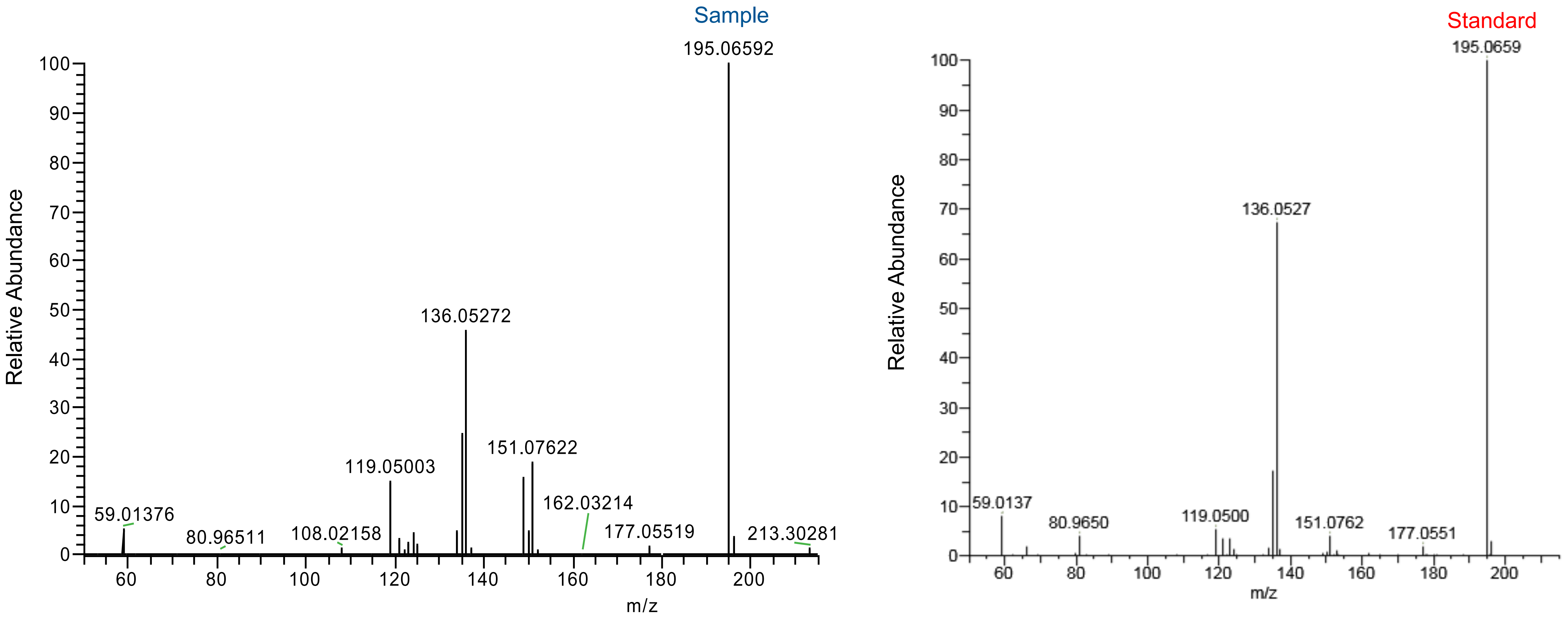

Supplementary Fig. 1-15: The secondary mass spectrogram of homoveratric acid. Left: sample; right: standard.

2,3-dihydroxybenzoate

Negative mode

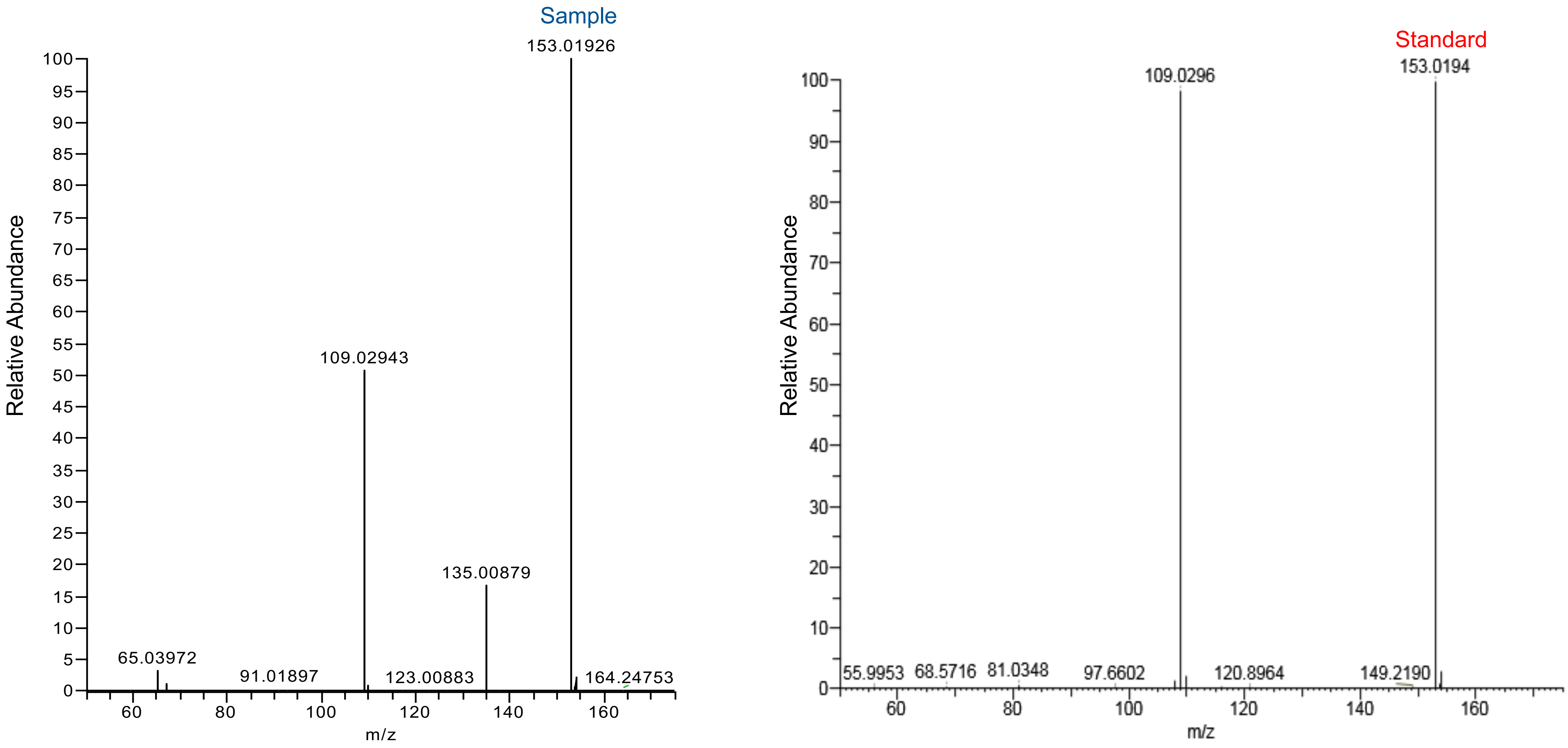

Supplementary Fig. 1-16: The secondary mass spectrogram of 2,3-dihydroxybenzoate. Left: sample; right: standard.

P-cresol sulfate

Negative mode

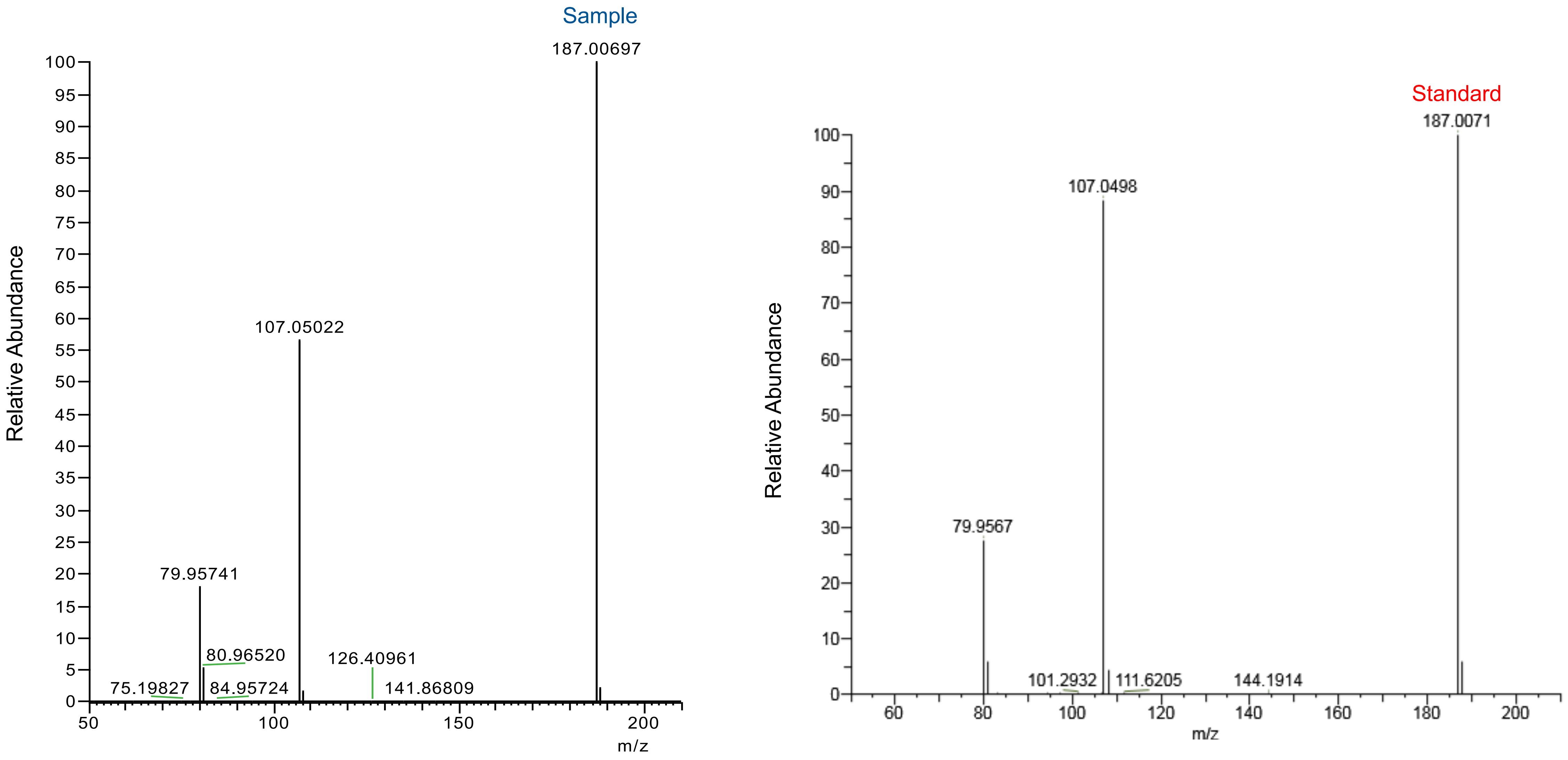

Supplementary Fig. 1-17: The secondary mass spectrogram of p-cresol sulfate . Left: sample; right: standard.

Trans-4-hydroxyproline

Negative mode

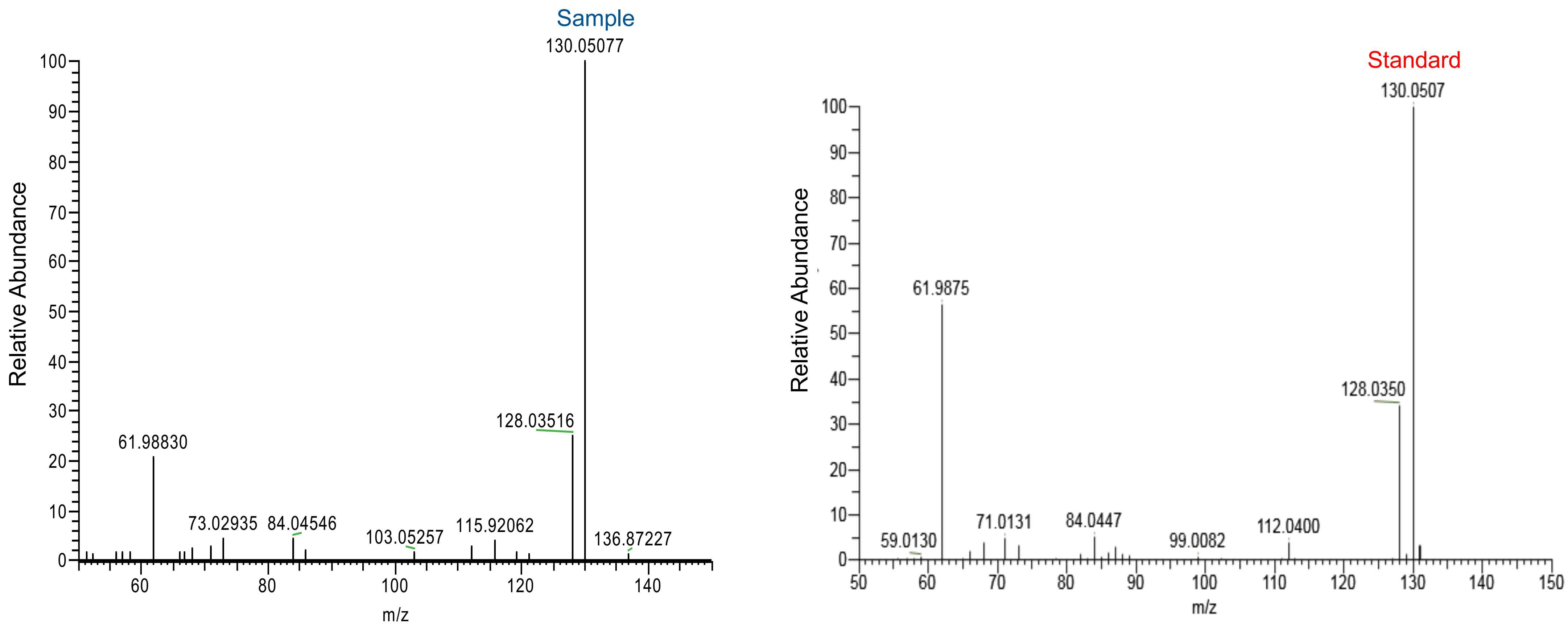

Supplementary Fig. 1-18: The secondary mass spectrogram of trans-4-hydroxyproline. Left: sample; right: standard.

Hippurate

Negative mode

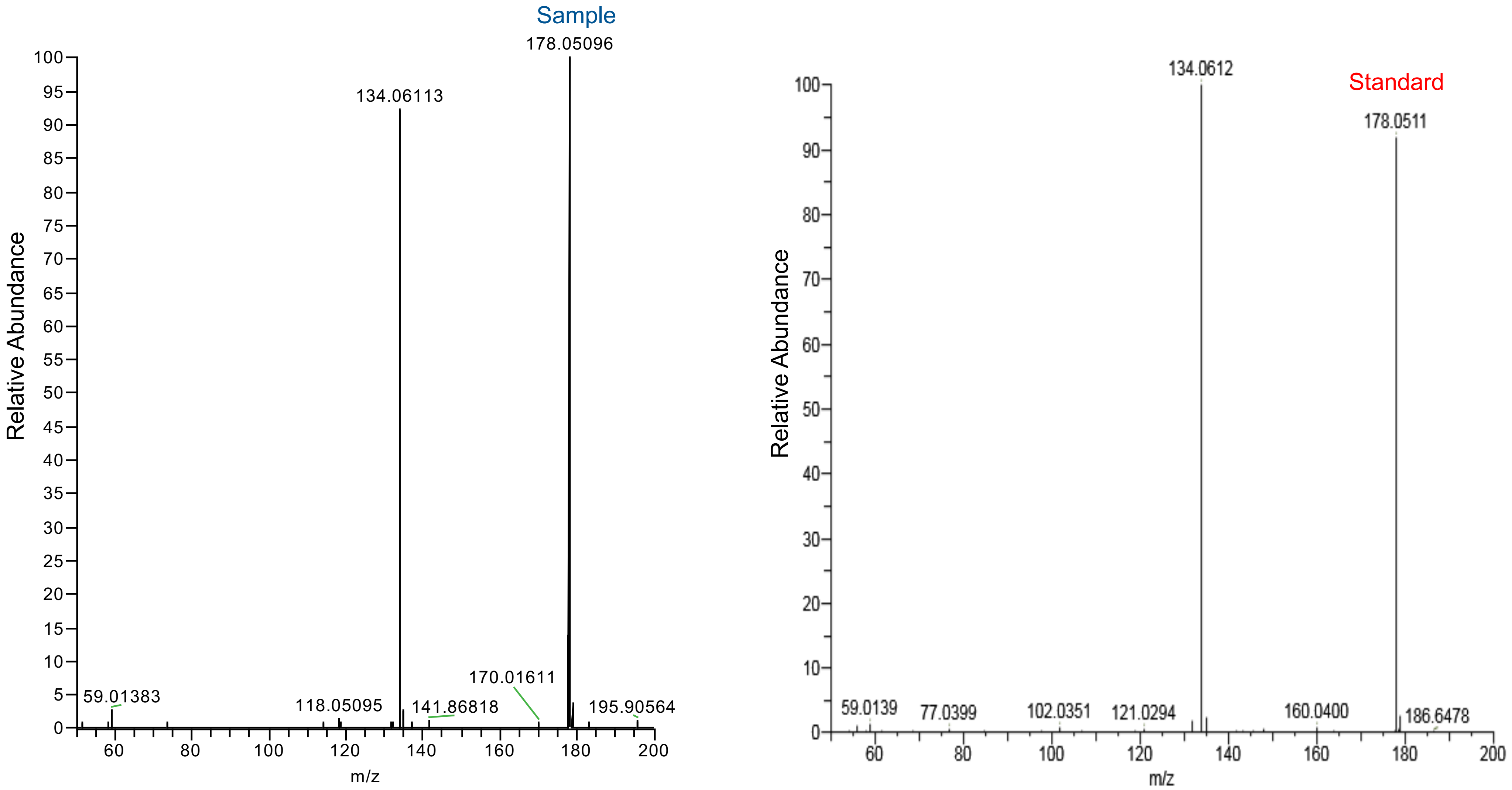

Supplementary Fig. 1-19: The secondary mass spectrogram of hippurate. Left: sample; right: standard.

Indoxyl sulfate

Negative mode

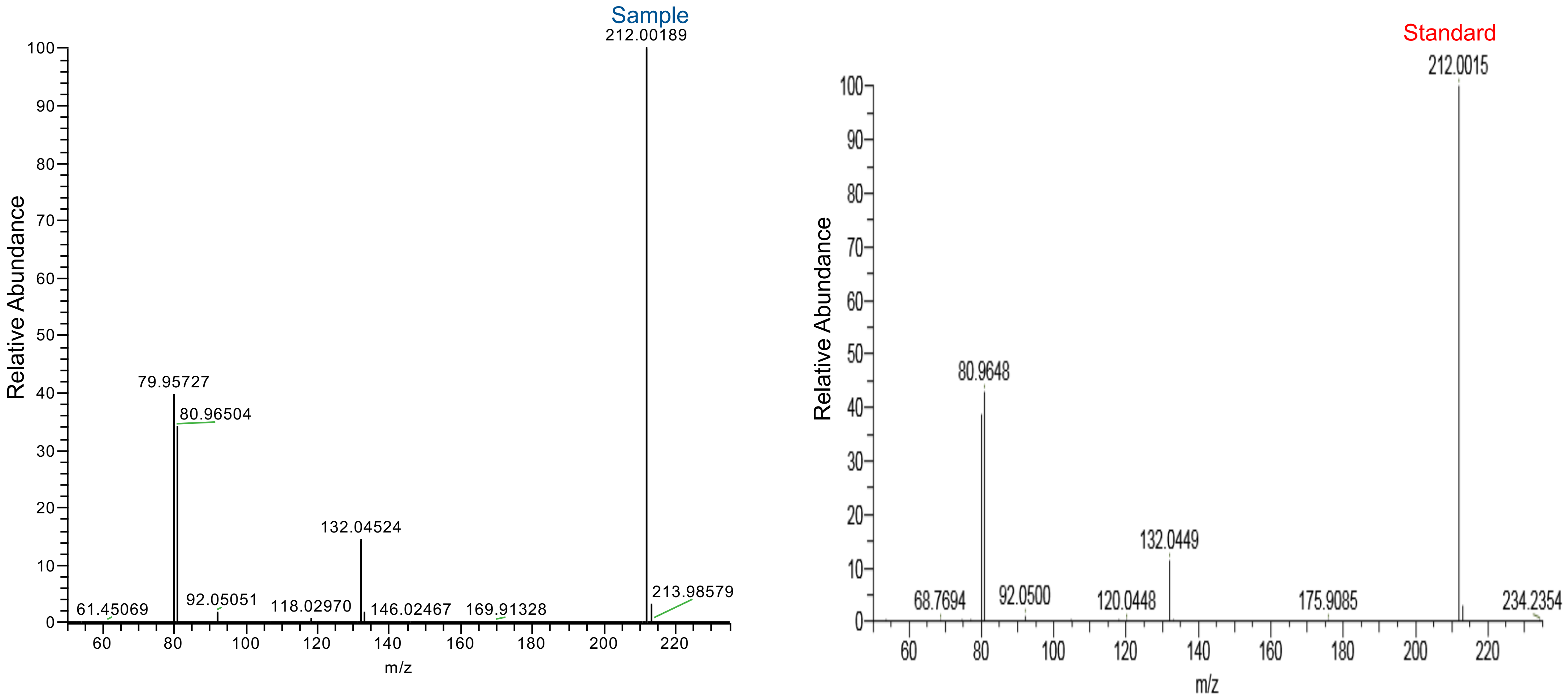

Supplementary Fig. 1-20: The secondary mass spectrogram of indoxyl sulfate. Left: sample; right: standard.

3-indoleacetic acid

Negative mode

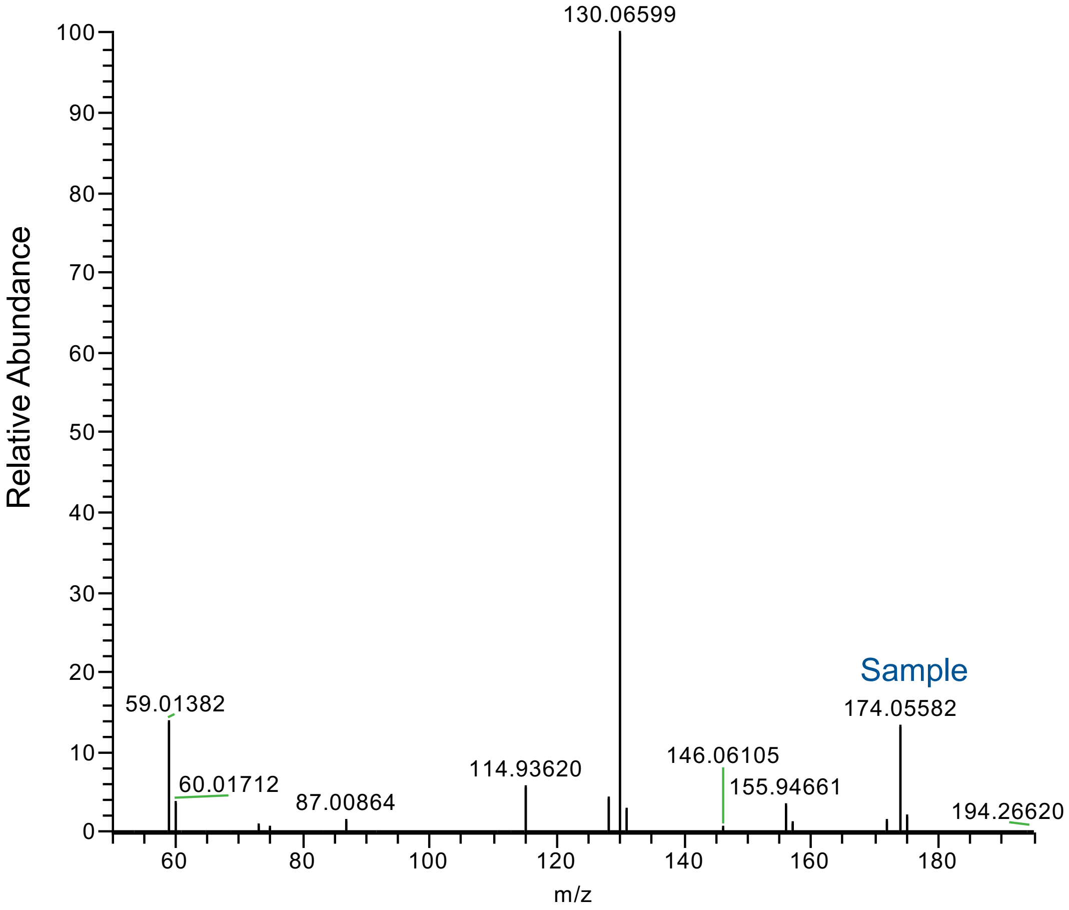

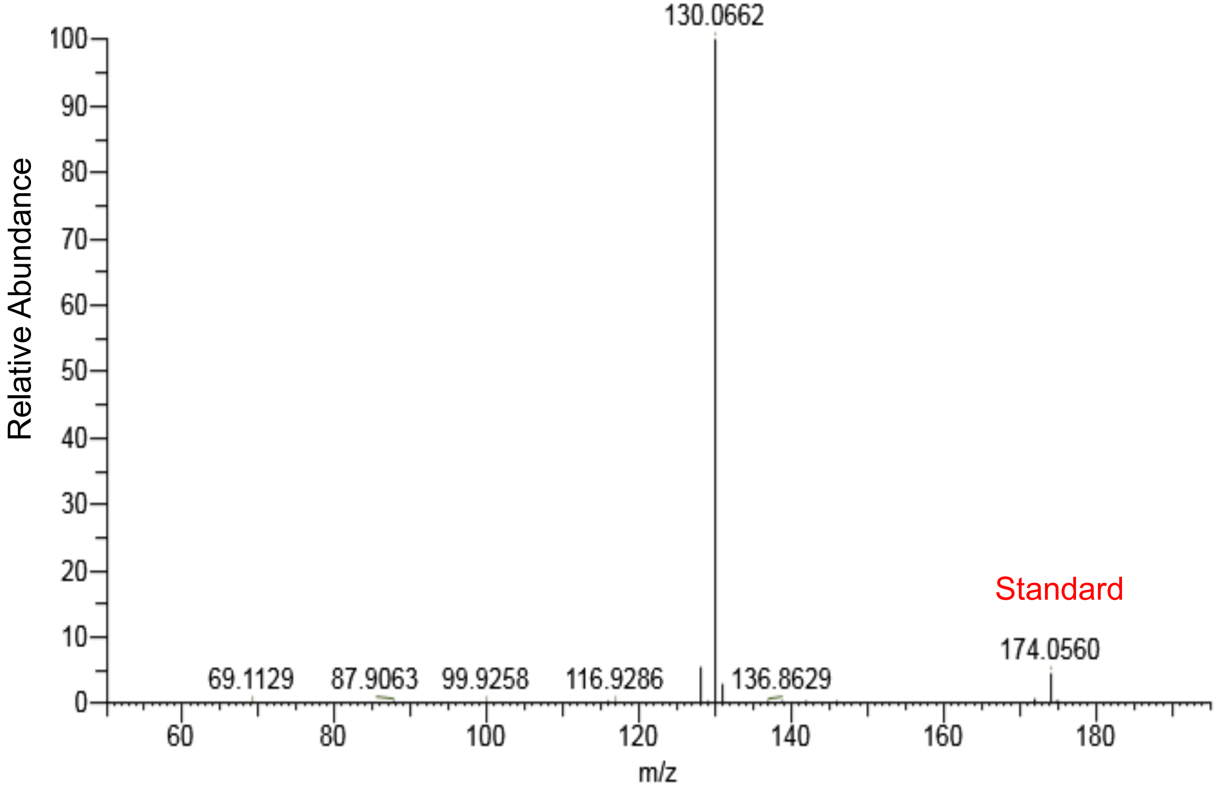

Supplementary Fig. 1-21: The secondary mass spectrogram of 3-Indoleacetic acid. Left: sample; right: standard.

LPE(18:2)-H

Negative mode

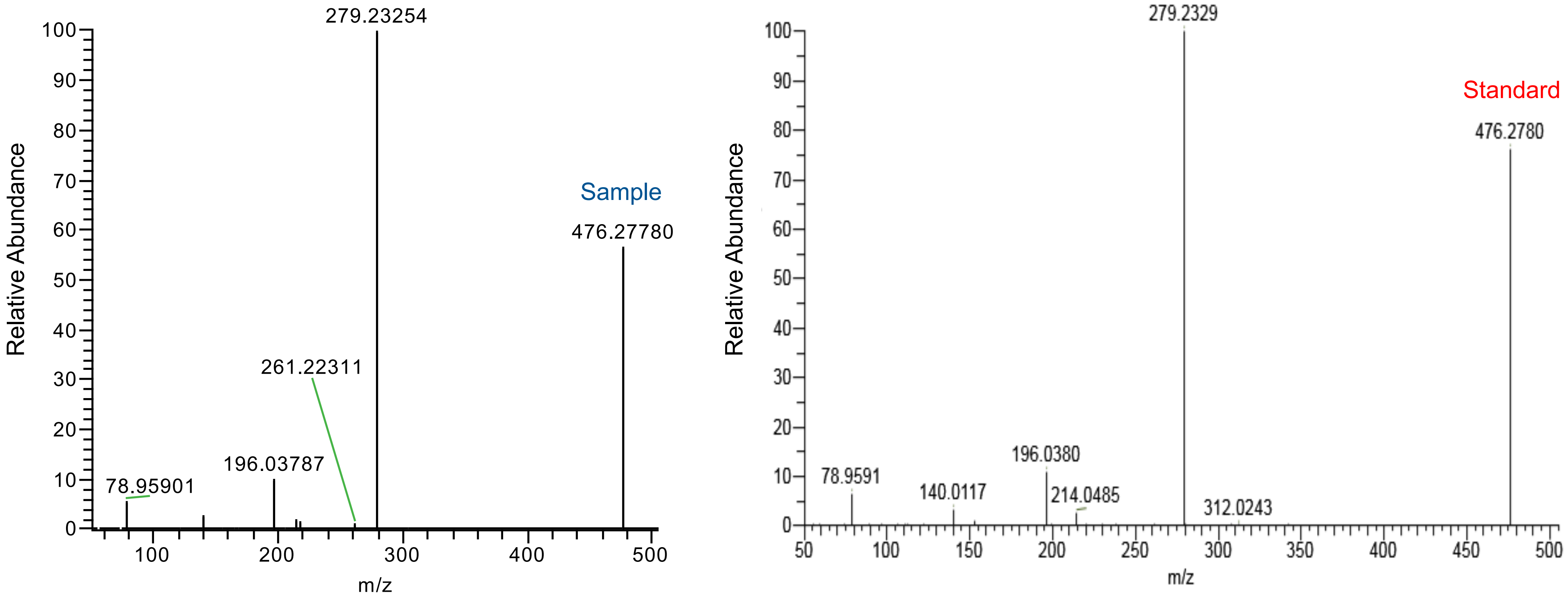

Supplementary Fig. 1-22: The secondary mass spectrogram of LPE(18:2)-H. Left: sample; right: standard.

4-pyridoxic acid

Negative mode

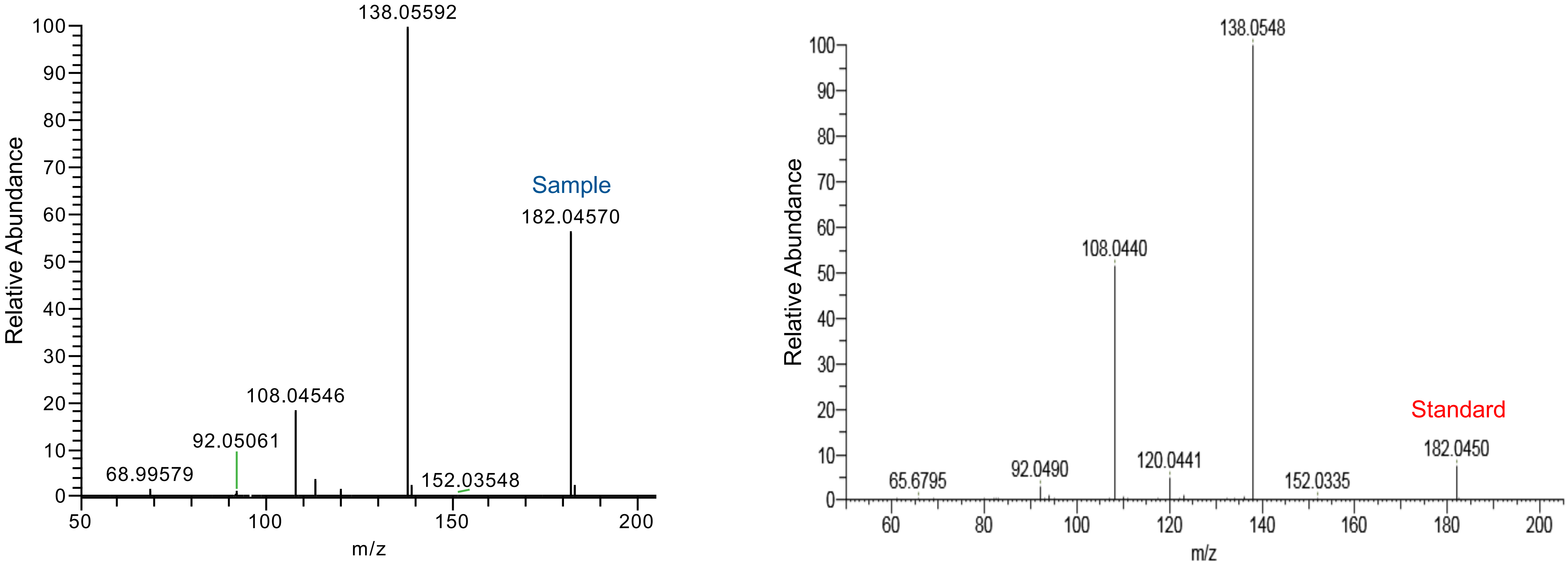

Supplementary Fig. 1-23: The secondary mass spectrogram of 4-pyridoxic acid. Left: sample; right: standard.
